# Fusing meteorological factors and baidu search index with an adapted deformtime model to predict influenza-like illness in Beijing

**DOI:** 10.3389/fpubh.2026.1856514

**Published:** 2026-09-03

**Authors:** Lingling Shen, Yajun Xiong, Zhaobin Sun

**Affiliations:** 1 Beijing Meteorological Data Center, Beijing, China; 2 Chinese Academy of Meteorological Sciences, Beijing, China

**Keywords:** baidu search index, Beijing, deep learning, DeformTime, epidemic early warning, influenza forecasting, influenza-like illness, meteorological factors

## Abstract

**Background:**

Influenza-like illness (ILI) poses a significant burden on public health systems, particularly in densely populated megacities. Accurate and timely forecasting is crucial for early warning and resource allocation. While deep learning models have shown promise, capturing the complex, non-linear coupling between environmental drivers and human behavior remains a challenge.

**Methods:**

We used weekly ILI% surveillance data from Beijing (2010–2019), complemented by 20 meteorological variables and 322 Baidu Search Keyword Indices. Cross-correlation function (CCF) analysis with seasonal differencing, ADF stationarity testing, and two-stage Benjamini–Hochberg FDR correction (*q* < 0.05) identified 11 predictive features, 6 meteorological variables and 5 search-derived features. An Adapted DeformTime model (DeformTime-Ad) was developed, incorporating a task-decoupled attention block, epidemic-level sample weighting, per-fold Optuna hyperparameter optimization, and Huber loss. Performance was evaluated against 10 comparators: persistence, seasonal-naïve, calendar-based, SARIMA, ARIMAX, SARIMAX, XGBoost, Random Forest, LSTM, and unadapted DeformTime-Base, under a 3-fold rolling-origin protocol across four horizons (7–28 days). Pairwise Diebold–Mariano tests, stratified evaluation by epidemic intensity, and six ablation variants were conducted.

**Results:**

Beijing ILI% exhibited a pronounced unimodal winter–spring peak. On common target weeks, DeformTime-Ad achieved the lowest MAE at 7-−14 days (0.39–0.45) and the lowest MSE at 7–21 days, representing up to 62.0% MSE reduction over a reporting-lag-corrected persistence benchmark. In Diebold–Mariano tests, DeformTime-Ad was significantly superior to 8, 6, 2, and 1 of 10 comparators at 7, 14, 21, and 28 days, respectively, with zero significant losses at any horizon. During epidemic peaks (5 common target weeks), DeformTime-Ad attained a peak MAE of 2.09, 31.9% lower than DeformTime-Base, though the persistence benchmark achieved the lowest peak MAE (0.78) on this small sample, reflecting high autocorrelation during sustained peak plateaus. Ablation analysis identified per-fold hyperparameter optimization as the most impactful component (+51.9% MSE when removed), followed among architectural components by the task-decoupled attention block (+7.9%); epidemic-level sample weighting improved peak-period accuracy at a small overall-MSE trade-off. Meteorological and search-index features provided complementary, horizon-dependent predictive signals.

**Conclusions:**

A multimodal deep-learning architecture with epidemic-aware design substantially improves weekly ILI% forecasting in Beijing, particularly at 1–3 week horizons. Both meteorological and internet-search features contribute predictive signal in a horizon-dependent manner, with meteorological variables gaining prominence at longer lead times. The framework, combining exploratory CCF-based feature screening, leakage-free rolling-origin evaluation on common target weeks, and component-level ablation, offers a methodology warranting further prospective, multi-city validation before operational deployment for infectious disease surveillance.

## 1 Introduction

Influenza-like illness (ILI) is a clinical syndrome used for surveillance and clinical screening of respiratory infectious diseases, rather than a single disease entity. In China, ILI is officially defined as acute onset of fever (body temperature ≥ 38 °C) accompanied by either cough or sore throat, as specified in the Guidelines for Response to Influenza-like Illness Outbreaks (2018 Edition) (1). The World Health Organization (WHO) defines ILI as an acute respiratory infection with onset within the past 10 days, accompanied by fever ≥ 38 °C and cough (2). As a common symptom cluster of respiratory infectious diseases, ILI serves as a core indicator for outbreak early warning and epidemiological surveillance of influenza and other related diseases.

ILI prediction is a critical component of respiratory infectious disease prevention and control. It enables the early judgment and early warning of epidemic trends, provides scientific support for optimizing medical resources and formulating precise prevention and control strategies, and is of great significance for improving the public health emergency response system.

In recent years, research on ILI prediction has evolved from single time-series modeling to a new stage of multi-source data fusion and mechanism analysis. Meteorological factors play a key role in this process and show significant regional heterogeneity. In temperate regions, cold and dry conditions are widely recognized as favorable for the survival and transmission of influenza viruses (3, 39). A global study across 57 countries further confirmed that influenza peaks in temperate regions are predominantly associated with cold-dry meteorological modes, whereas tropical regions exhibit warm-humid modes, with a temperature transition threshold of approximately 16.5–19.5 °C (4). In contrast, tropical and subtropical regions exhibit more complex patterns: rainfall may indirectly enhance transmission by promoting indoor crowding, with surface temperature as the dominant driver (5). A large-scale study covering 339 cities in China found that each 1 °C increase in short-term temperature variability was associated with a 3.3% rise in influenza incidence, and this effect was significantly amplified under high PM_2_.5 pollution conditions, especially among older adults in cold seasons in northern China (6). Existing studies have shown that influenza in temperate regions displays a single winter peak (7), with temperature and humidity as major influencing factors for influenza occurrence and transmission (8–13). In contrast, influenza in southern China mostly shows a bimodal pattern (7, 14–16).

Early studies on ILI prediction relied mostly on univariate time series models such as SARIMA, which featured clear structures but were limited in capturing nonlinear dynamics (17). Google Flu Trends, the world's first influenza surveillance system based on big search data launched by Google in 2008, established a new paradigm for disease monitoring using search data and steered ILI prediction research toward multimodal data fusion. Online search behaviors represented by the Baidu Index have been proven to show a 1–3 week leading lag for ILI, and their significance has been quantified via cross-correlation function analysis (18). Santillana et al. (19) proposed an ensemble machine learning framework for influenza surveillance that integrated five data sources, including Google search queries, Twitter data, and near-real-time outpatient records to achieve real-time nowcasting and up to 3 weeks of short-term ILI forecasting in the United States. Wang et al. (20) integrated sentinel surveillance data, search indices of 27 keywords, and 8 meteorological variables to develop a dynamically weighted deep learning ensemble framework for ILI prediction in Yangzhou, reducing RMSE by 27.9% compared with the best single model. A study by Orang et al. (17) on Canadian ILI indicated that although SARIMA achieved higher overall accuracy, artificial neural networks showed superior performance in capturing peak weeks of influenza epidemics.

Although existing studies have explored the association between online search data and influenza prediction, systematic integration of meteorological data, search behavior data, and ILI clinical data targeting megacities such as Beijing remains limited (21, 22). Integrating multi-source data within a deep learning model framework for ILI prediction continues to be a research frontier in the digital transformation of public health (23, 24). As a megacity with distinct temperate continental climate characteristics, the coupled impacts of meteorological conditions and public behavioral responses on ILI dynamics in Beijing have not yet been systematically investigated across multiple forecast horizons.

Therefore, this study adopts DeformTime, a novel multivariate time-series forecasting deep learning model proposed by Shu and Lampos (25), and customizes it for Beijing by incorporating meteorological factors, Baidu Search Index data, and local epidemiological characteristics. We systematically compare the adapted model against ten benchmarks, persistence, seasonal-naïve, calendar-based, SARIMA, ARIMAX, SARIMAX, XGBoost, Random Forest, LSTM, and unadapted DeformTime-Base, across forecast horizons of 7–28 days using three-fold rolling-origin cross-validation, with stratified evaluation by epidemic activity level. This study aims to provide a spatiotemporally accurate forecasting methodology that warrants further prospective validation for influenza prevention and control in temperate megacities.

## 2 Data and methods

### 2.1 Data

The meteorological data used in this study comprise daily observation data from 2010 to 2019, obtained from the National Basic Meteorological Elements Daily Dataset (V3.0) of China's national ground meteorological stations. Daily data were aggregated to weekly meteorological factors including mean, maximum, minimum, and sum values, as described in Table 1.

**Table 1:** Processing of daily meteorological variables into weekly indices.

Aggregation	Variables
Mean	Weekly mean temperature, weekly mean wind speed, weekly mean relative humidity
Maximum	Weekly maximum temperature, weekly maximum temperature diurnal range, weekly maximum wind speed, weekly extreme wind speed, weekly maximum air pressure
Minimum	Weekly minimum temperature, weekly minimum relative humidity, weekly minimum air pressure
Sum	Weekly total precipitation, weekly sunshine duration, occurrence counts of floating dust, strong wind, sandstorm, fog, snow, and haze events; weekly cold air frequency index

The weekly cold air frequency index was calculated following the national standard Grades of Cold Air (GB/T 20484-−2017) (38). Cold air events are classified into four grades (weak, moderately strong, strong and cold wave) based on 24-h or 48-h cooling amplitude of minimum temperature. Weights for each grades are derived via information entropy weighting, and the weekly index is obtained by summing the daily weighted values.

ILI data comprise the weekly ILI case counts (ILI_count, the total number of ILI consultations at sentinel hospitals per week) and ILI% (the ratio of ILI consultations to total outpatient visits) in Beijing from 2010 to 2019.

ILI% observations were stratified into three activity levels independently within the training portion of each fold, with no access to test-period data. For each fold, epidemic activity levels are defined using the training-set mean and standard deviation of ILI% (Table 2). This approach is consistent with established statistical thresholding frameworks in epidemiological time-series surveillance (26). Statistical analysis of ILI% data reveal severe sample imbalance across the three activity levels of ILI% (Supplementary Table S0). Sample weighting is therefore adopted in this study to assign distinct weights to the three activity levels, whereby a higher weight was allocated to the peak level with a smaller sample size.

**Table 2:** ILI% activity–level thresholds, epidemiological meaning, and weights.

Level	Threshold	Epidemiological meaning	weight
Non–epidemic	ILI% < mean + 1 SD	Baseline transmission level	1.0
Active	mean + 1 SD ≤ ILI% < mean + 2 SD	Elevated activity	2.0
Peak	ILI% ≥mean + 2 SD	High–intensity outbreak	3.0

Online search data were obtained from the Baidu Index open platform (https://index.baidu.com), comprising daily search indices for 322 Chinese keywords related to influenza symptoms, prevention, healthcare-seeking, and treatment during 2010–2019. Daily keyword indices were aggregated to weekly totals for model input. Keywords are classified into four categories (Table 3).

**Table 3:** Classification and description of baidu search index keywords.

Category	Count	Representative keywords
Prevention	10	Flu shot(流感疫苗), H1N1 prevention (甲流预防)
Symptom	105	Cold(感冒), H1N1 flu(甲流), Fever(发烧), Cough(咳嗽), Sore throat(咽痛)
Healthcare–seeking	10	Which department for cold(感冒挂什么科), Fever clinic(发热门诊)
Treatment Intervention	197	Oseltamivir(奥司他韦), Contac NT(新康泰克), Foods for fever(发烧吃什么食物好)

In total, 20 meteorological variables and 322 Baidu Search Index variables were used, covering the period 2010-−2019. The dataset was split chronologically without shuffling into a three-fold rolling-origin evaluation framework: Fold 1 (train: 2010–2016, test: 2017), Fold 2 (train: 2010–2017, test: 2018), and Fold 3 (train: 2010–2018, test: 2019). Within each fold, the last year of the training set was held out for hyperparameter validation, and all data-dependent procedures (scaling, threshold estimation, sample weighting, and model training) were performed exclusively on the training portion of that fold. CCF analysis was conducted exclusively on the training set (2010–2016) to prevent data leakage. Robust scaling (median and interquartile range estimated from the training set) was applied to meteorological variables, and min–max normalization (minimum and range estimated from the training set) was applied to Baidu Search Index variables, with parameters fixed and applied to the validation and test sets without re-fitting.

### 2.2 Methods

#### 2.2.1 Cross-correlation function of variables

The cross-correlation function (CCF) is a core statistical tool for quantifying the strength and direction of linear correlation between two time series at different time lags. Its core value lies in revealing potential temporal dependencies between variables, providing a critical basis for time series modeling and forecasting (27).

For stationary time series *Xt* and *Yt*, the CCF can be calculated using the following formula:


$$\begin{array}{l} CCF\left(\tau \right)=\frac{Cov\left(Xt,Yt+\tau \right)}{\sqrt{Var\left(Xt\right)\cdot Var\left(Yt\right)}} \end{array}$$ (1)

where τ denotes the lag order (*X* leads *Y* when τ>0; *Y* leads *X* when τ < 0), *Cov*(*Xt, Yt*+τ) is the covariance between the two series at lag τ, and *Var*(*Xt*) and *Var*(*Yt*) are the variances of the two series, respectively. The value range of CCF is [−1,1]; the closer the absolute value is to 1, the stronger the linear correlation at the corresponding lag.

The ILI%, meteorological, and search index series were first rendered stationary through seasonal differencing and ADF testing. After aligning differencing orders, CCF were computed over a lag range of −4 to +4 weeks. A two-stage Benjamini–Hochberg false discovery rate (BH-FDR) procedure was applied to control for multiple comparisons, and CCF morphology quality control was used to screen candidate features. Only variables that were FDR-significant with a non-negative optimal lag were retained as forecasting candidates. The selected Baidu search variables were grouped according to the categories in Table 3, large categories were subjected to principal component analysis (PCA) to construct composite search indices, whereas small categories retained their top-correlated keywords as standalone features. Meteorological variables were kept in their original form to preserve their physical interpretability. Details of the CCF computation are provided in Supplementary Text S1. The CCF analysis is used as an exploratory feature-screening procedure; *q*-values are descriptive and should not be interpreted as confirmatory inference, as the procedure does not fully account for data-driven lag selection or residual serial dependence (prewhitening not performed).

#### 2.2.2 Model algorithms

Shu and Lampos (25) proposed DeformTime, a novel deep learning architecture for multivariate time series forecasting. It captures cross-variable temporal dependencies via the Variable Deformation Attention Block (V-DAB) and within-variable temporal dependencies via the Temporal Deformation Attention Block (T-DAB), combined with Neighborhood-Aware Input Embedding (NAE) and dual positional encoding. The model demonstrated outstanding performance on benchmark datasets (ETTh1, ETTh2, Weather) and ILI forecasting tasks in England and the United States, while exhibiting low GPU memory consumption (25).

**Figure 1:**
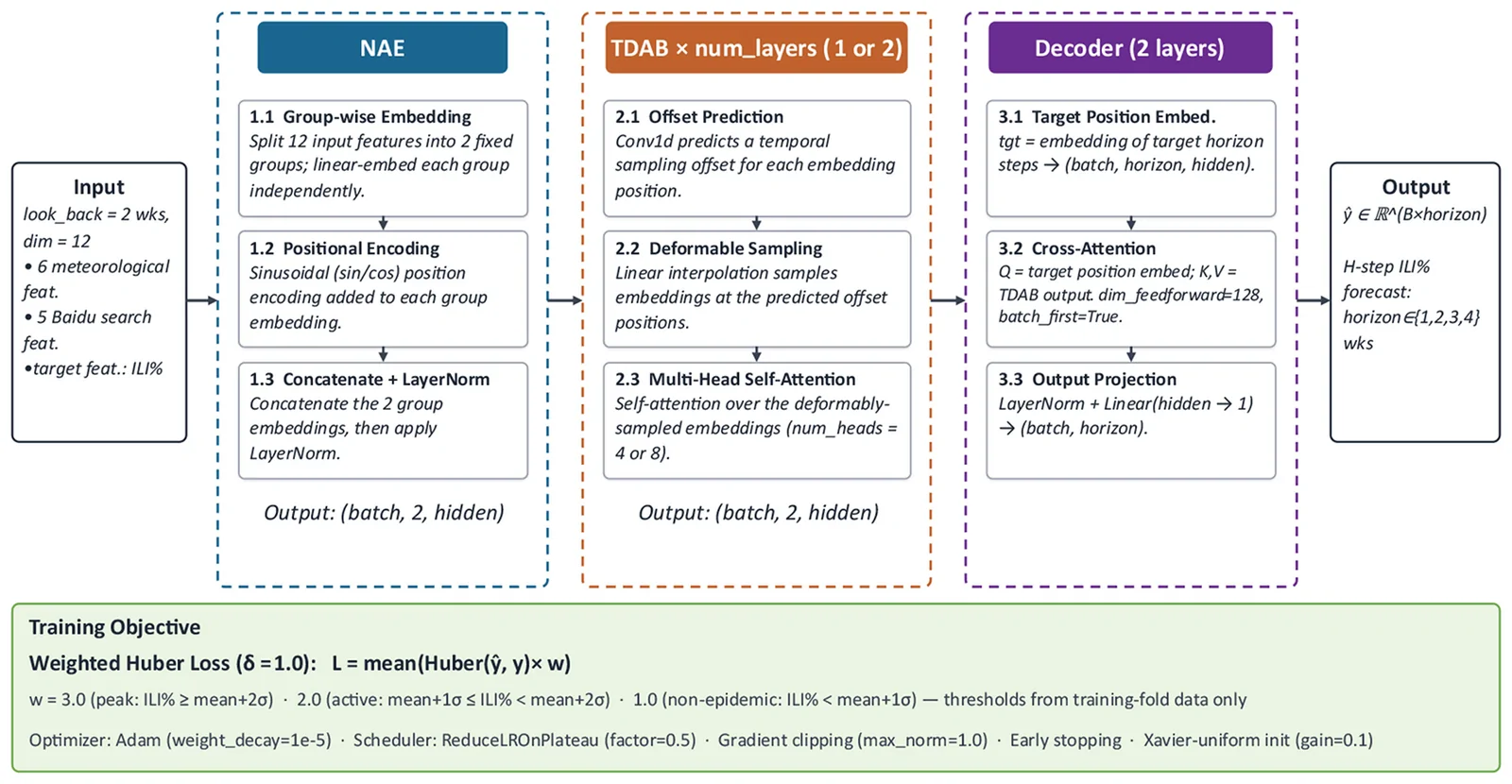
Architecture of the adapted DeformTime model.

Original DeformTime is designed for long-sequence, large-scale multivariate time series forecasting, with default configurations (e.g., d_model = 512) tailored to datasets containing tens of thousands of hourly observations. Given the practical constraints of our ILI surveillance data, limited sample size, short lookback window, and moderate feature dimensionality, directly applying the official architecture would lead to severe over-parameterization. We therefore adopt a DeformTime-inspired lightweight architecture, termed DeformTime-Base, which preserves the core idea of deformable temporal attention while replacing the heavy CrossDeformAttn module with a streamlined NAE and TDAB. Building upon DeformTime-Base, our Adapted DeformTime further introduces: (i) convolutional offset prediction in the TDAB replacing gather-based deformable sampling, (ii) Bayesian hyperparameter optimization, (iii) a sample weighting scheme that assigns more weight to peak weeks' data to address class imbalance, and (iv) MSE loss function was replaced with Huber loss to achieve greater robustness against ILI% spikes (Figure 1).

Several additional models were included for comparison. LSTM (Long Short-Term Memory) is an improved recurrent neural network that addresses the vanishing gradient problem through gating mechanisms and cell states, enabling capture of long-term temporal dependencies in sequential data (28). Random Forest is a decision tree ensemble that reduces overfitting via bootstrap aggregation and random feature selection, exhibiting good noise resistance and the ability to rank feature importance without requiring complex preprocessing (29, 30). XGBoost is an optimized gradient boosting implementation that iteratively fits residuals, incorporates regularization and employs column subsampling and parallel computing to improve generalization in small-sample, high-dimensional prediction tasks (31). SARIMA, ARIMAX, and SARIMAX are classic Box–Jenkins time-series models. SARIMA incorporates seasonal differencing to capture periodic epidemic fluctuations (32). ARIMAX extends the non-seasonal ARIMA framework with external explanatory covariates. SARIMAX simultaneously integrates seasonal structures and exogenous predictors, providing robust predictions for seasonally modulated outcomes (33).

To evaluate the contribution of each modification in the Adapted DeformTime model, six ablation experiments were conducted. Four structural ablations each removed a single improvement from the Adapted DeformTime: (1) TDAB module (Conv1d-offset with interpolation versus Linear-offset with gather), (2) hyperparameter optimization (Optuna-based search versus fixed default parameters), (3) sample weighting (epidemic-level weights versus uniform weighting), and (4) loss function (HuberLoss versus MSELoss). Two additional feature-level ablations were designed to quantify the relative contributions of meteorological and search-index variables: (5) meteorological-only and (6) search-only.

For benchmark comparison, three naïve reference models were implemented under the identical evaluation framework: Persistence (Y(t+h) = Y(t−1)), Seasonal-naïve (Y(t+h) = Y(t−52)), and Calendar-based (linear regression on Fourier harmonic features of order 3). In China's ILI surveillance system, weekly ILI% data are compiled from sentinel hospitals with a reporting lag of approximately 1 week, the value for week t is typically not available until week t+1. At the forecast origin (week t), the most recent available ILI% observation is therefore Y(t−1), not Y(t). The persistence benchmark accordingly uses Y(t−1) as the predicted value, consistent with the information genuinely available at the forecast origin.

In addition, SARIMA, ARIMAX, and SARIMAX were included as classical time-series baselines, while XGBoost and Random Forest served as tree-based machine-learning baselines and LSTM served as a deep-learning baseline.

The SARIMA model without exogenous variables also functioned as a seasonal autoregressive benchmark. For ARIMAX and SARIMAX, which require exogenous regressors at future time steps, last-known-value persistence was employed (i.e., $\hat{\text{X}}$(t+k) = X(t) for all *k* > 0), ensuring that no future observed values of meteorological or search-index data were used in multi-step forecasting.

The supervised models' use of [Y(t−2), Y(t−1)] as the lookback window reflects the same reporting lag, whereas the exogenous variables (meteorological data and Baidu search indices) have much shorter reporting delays, so X(t) is legitimately available at the forecast origin.

To ensure fair comparison, all models were evaluated under the same three-fold rolling-origin cross-validation framework with four forecast horizons (7, 14, 21, and 28 days).

Models incorporating exogenous variables shared the same CCF-selected feature set and per-fold feature scaling, with scalers fitted exclusively on each fold's training data.

The direct multi-step forecasting convention was: $\hat{\text{Y}}$(t+h) = f(X (t), Y(t−2), Y(t−1)), where h is the forecast horizon in weeks (h = 1–4 for 7–28 day horizons), X (t) denotes the 11 CCF-selected features at week t, and Y(t−2), Y(t−1) are the two preceding weekly ILI% observations (lookback = 2 weeks). For example, to forecast ILI% at the 7-day horizon (h = 1), the model uses features from week t and ILI% from weeks t−2 and t−1 to predict ILI% at week t+1; for the 28-day horizon (*h* = 4), the same inputs are used to predict ILI% at week t+4.

The lookback window was uniformly set to 2 weeks across all other models, except SARIMA, ARIMAX, SARIMAX.

Hyperparameter optimization was performed using Optuna for DeformTime-Ad, XGBoost, Random Forest, and LSTM models; SARIMA, ARIMAX, and SARIMAX employed grid search for order selection owing to their smaller parameter spaces; the DeformTime-Base ablation used fixed default parameters for direct structural comparison.

Epidemic-level sample weighting was applied to the Adapted DeformTime, XGBoost, Random Forest, and LSTM models, as these frameworks support instance-level weighting; SARIMA, ARIMAX, SARIMAX, and the naïve benchmarks do not support instance-level weighting and were therefore trained without sample weights.

All models were evaluated on the same unweighted metrics (MAE, SMAPE, MSE) to ensure a fair comparison.

#### 2.2.3 Evaluation metrics and validation design

Three metrics were used to evaluate prediction accuracy: Mean Absolute Error (MAE), Mean Squared Error (MSE), and Symmetric Mean Absolute Percentage Error (SMAPE) (34).

MAE measures average absolute deviation and is robust to outliers (35). Its formula is:


$$\begin{array}{l} MAE=\frac{1}{n}\sum_{i=1}^{n}|y_{i}-{ŷ}_{i}| \end{array}$$ (2)

MSE penalizes large errors more heavily. Its formula is:


$$\begin{array}{l} MSE=\frac{1}{n}\sum_{i=1}^{n}{\left(y_{i}-{ŷ}_{i}\right)}^{2} \end{array}$$ (3)

SMAPE provides a unit-free relative measure that avoids distortion near zero. Its formula is:


$$\begin{array}{l} SMAPE=\frac{100\%}{n}\sum_{i=1}^{n}\frac{\left|y_{i}-{ŷ}_{i}\right|}{\left(\left|y_{i}|+|{ŷ}_{i}\right|\right)/2} \end{array}$$ (4)

In the above formulas, *y*_*i*_ is the observed value, ŷ_*i*_ is the predicted value, and *n* is the number of samples.

Due to differences in model architecture, the effective evaluation window differs across model families. SARIMA-based models employ an expanding-window protocol, the model is fit once on the training set and the Kalman-filter state is updated at each forecast origin without parameter refitting, generating predictions for all 52 weeks of each test year. DeformTime-based models use a sliding-window approach with a fixed 2-week burn-in; with horizon-specific output-step extraction, the native evaluation window spans 50, 49, 48, and 47 weeks at the 7-, 14-, 21-, and 28-day horizons, respectively. Supervised learning models require a horizon-dependent burn-in period of lookback + horizon weeks (3–6 weeks for 7–28 day horizons, yielding first predictions from weeks 4–7).

To ensure fair comparison, all main comparative metrics (Tables 4–7) are computed on common target weeks, the intersection of target weeks available across all 11 model families for each horizon (*n* = 147, 144, 141, and 138 at 7, 14, 21, and 28 days, respectively). Native-window metrics are retained as a secondary analysis in Supplementary Table S10. We note that the per-year array segmentation in our implementation results in an additional 2-week burn-in (equal to the lookback window) for supervised models, beyond the horizon-dependent burn-in inherent to the direct multi-step framework; historical observations from the preceding year could in principle be used to construct lookback sequences for the first weeks of each test year. This implementation choice does not affect the fairness of the common-target-week comparison, as all models are evaluated on identical target weeks.

**Table 4:** Per-horizon forecasting performance of all models (3-fold rolling-origin evaluation, common target weeks).

Horizon	7–day horizon (*N* = 147)			14–day horizon (*N* = 144)			21–day horizon (*N* = 141)			28–day horizon (*N* = 138)		
Model	MAE	SMAPE (%)	MSE	MAE	SMAPE (%)	MSE	MAE	SMAPE (%)	MSE	MAE	SMAPE (%)	MSE
Persistence	0.555	13.89	0.844	0.696	17.84	1.233	0.764	19.41	1.487	0.887	22.53	1.891
Seasonal–naïve	0.792	22.52	1.223	0.78	22.48	1.175	0.788	22.79	1.191	0.726	21.86	0.895
Calendar	0.714	19.9	0.831	0.689	19.64	0.769	0.689	19.78	0.774	0.659	19.4	**0.637**
SARIMA	0.474	12.75	0.539	0.529	14.07	0.675	**0.543**	**14.62**	0.714	**0.600**	17.26	0.687
ARIMAX	0.57	16.15	0.578	0.607	17.26	0.689	0.689	19.55	0.878	0.635	18.82	0.682
SARIMAX	0.572	16.23	0.598	0.631	17.89	0.751	0.728	20.62	0.985	0.672	19.82	0.776
XGBoost	0.571	15.82	0.608	0.651	18.33	0.735	0.675	18.44	0.936	0.753	21.22	0.909
Random Forest	0.501	13.45	0.537	0.613	16.76	0.748	0.669	18.01	0.946	0.745	20.66	1.031
LSTM	0.68	18.71	0.917	0.732	20.69	0.975	0.672	19.14	0.854	0.645	18.88	0.647
DeformTime–Base	0.531	14.78	0.557	0.56	15.77	0.584	0.642	18.15	0.73	0.671	19.46	0.727
DeformTime–Ad	**0.392**	**10.55**	**0.394**	**0.451**	**12.16**	**0.468**	0.561	15.21	**0.699**	0.611	**17.17**	0.732

Bold indicates the best value per horizon.

**Table 5:** Diebold–Mariano test results, significant wins and losses by model and horizon.

Model	7d W/L/Net	14d W/L/Net	21d W/L/Net	28d W/L/Net
Persistence	0/1/−1	0/5/−5	0/2/−2	0/3/−3
Seasonal–naïve	0/7/−7	0/5/−5	0/2/−2	0/1/−1
Calendar	0/5/−5	1/2/−1	0/0/0	0/0/0
SARIMA	3/0/+3	2/0/+2	2/0/+2	1/0/+1
ARIMAX	2/1/+1	2/0/+2	1/0/+1	0/0/0
SARIMAX	1/1/0	0/0/0	0/2/−2	0/0/0
XGBoost	1/1/0	0/1/−1	0/0/0	0/0/0
Random Forest	2/0/+2	1/1/0	0/0/0	0/0/0
LSTM	0/3/−3	0/2/−2	0/0/0	1/0/+1
DeformTime–Base	3/1/+2	4/0/+4	1/0/+1	1/0/+1
DeformTime–Ad	**8/0/+8**	**6/0/+6**	**2/0/+2**	**1/0/+1**

Each cell shows the number of comparator models that the row model significantly outperforms (Wins), is significantly outperformed by (Losses), and the net difference (Net = Wins – Losses). DM test uses MSE loss with Newey–West HAC standard errors, α = 0.05, two–sided. Out of 10 pairwise comparisons per model per horizon. DeformTime–Ad was never significantly outperformed by any model at any horizon (0 losses). The number of significant wins decreased at longer horizons, consistent with the MSE convergence observed in Table 4. Full *p*–value matrices are provided in Supplementary Table S4. The bold row highlights the results of the proposed DeformTime-Ad model.

**Table 6:** Stratified evaluation by epidemic intensity (4–horizon average, common target weeks, data–driven thresholds).

Period	Peak				Active				Non–epidemic			
Model	MAE	SMAPE(%)	MSE	*n*	MAE	SMAPE(%)	MSE	*n*	MAE	SMAPE(%)	MSE	*n*
DeformTime–Ad	2.09 ± 1.10	27.78 ± 14.19	5.49 ± 3.55	5	**1.03** **±0.68**	**21.39** **±14.99**	**1.62** **±1.44**	36	**0.45** **±0.11**	**13.02** **±3.00**	0.45 ± 0.19	529
Persistence	**0.78** **±0.60**	**9.53** **±7.71**	**0.88** **±0.94**	5	1.86 ± 0.45	34.87 ± 9.93	4.04 ± 1.52	36	0.65 ± 0.22	17.37 ± 4.52	1.20 ± 0.94	529
Seasonal–naïve	3.51 ± 0.90	53.26 ± 12.87	13.80 ± 4.85	5	2.14 ± 0.63	48.85 ± 15.00	5.45 ± 2.13	36	0.66 ± 0.10	20.49 ± 3.44	0.74 ± 0.23	529
Calendar	3.43 ± 1.02	50.35 ± 12.59	12.74 ± 6.39	5	1.34 ± 0.16	28.17 ± 4.14	2.19 ± 0.66	36	0.62 ± 0.11	18.81 ± 3.98	0.54 ± 0.07	529
DeformTime–Base	3.07 ± 0.60	44.37 ± 6.58	9.92 ± 3.44	5	1.47 ± 0.50	31.08 ± 11.45	2.53 ± 1.40	36	0.52 ± 0.08	15.85 ± 2.89	**0.44** **±0.12**	529
SARIMA	1.83 ± 0.98	23.78 ± 12.39	4.69 ± 3.04	5	1.44 ± 0.61	29.77 ± 14.18	2.56 ± 1.83	36	0.47 ± 0.09	13.60 ± 2.79	0.49 ± 0.30	529
SARIMAX	2.12 ± 0.82	28.35 ± 9.65	5.52 ± 3.07	5	1.27 ± 0.71	26.78 ± 16.18	2.25 ± 1.93	36	0.59 ± 0.14	17.92 ± 4.48	0.63 ± 0.29	529
ARIMAX	2.23 ± 0.95	30.12 ± 11.78	6.11 ± 3.85	5	1.26 ± 0.71	26.49 ± 16.10	2.19 ± 1.86	36	0.57 ± 0.13	17.17 ± 4.53	0.55 ± 0.25	529
XGBoost	2.31 ± 0.86	31.46 ± 10.45	6.30 ± 3.56	5	1.13 ± 0.67	23.39 ± 14.48	1.81 ± 1.58	36	0.62 ± 0.17	18.00 ± 4.69	0.68 ± 0.37	529
RandomForest	2.08 ± 1.01	27.85 ± 13.12	5.27 ± 4.41	5	1.15 ± 0.73	23.92 ± 16.46	1.99 ± 1.87	36	0.58 ± 0.15	16.64 ± 3.36	0.70 ± 0.43	529
LSTM	4.42 ± 0.91	70.83 ± 11.32	20.39 ± 7.90	5	2.02 ± 0.25	43.84 ± 5.14	4.23 ± 0.92	36	0.56 ± 0.13	17.24 ± 4.53	0.45 ± 0.16	529

^a^ 4 horizons average(mean ± SD across fold × horizon combinations with weeks in that epidemic level). Sample sizes: Peak *n* = 5 (3 at 7d, 1 at 14d, 1 at 21d, 0 at 28d; 28d excluded from peak average), Active *n* = 36, Non–epi *n* = 529, pooled across 3 folds × 4 horizons on common target weeks. Peak weeks derive from Fold 2 (2018) and Fold 3 (2019) only; Fold 1 (2017) had no peak weeks (max ILI% 6.34 < peak threshold 6.385). The very limited peak sample (*n* = 5) constrains the statistical reliability of the peak–period ranking. sMAPE uses NaN/Inf protection (denominator = 0 → 0%). Native–window stratified results (larger peak sample) are provided in Supplementary Table S11. Bold values indicate the lowest (best) error for each metric within each epidemic-intensity stratum.

**Table 7:** Ablation study results (4-horizon average, common target weeks, mean ± SD across 3 folds × 4 horizons).

Variant	Modification	MAE	SMAPE (%)	MSE	ΔMSE (%)	Peak MAE	Peak MSE
Full (DeformTime–Ad)	—	0.504 ± 0.119	13.77 ± 3.09	0.573 ± 0.236	—	2.09 ± 1.10	5.49 ± 3.55
Abl−1 (no Conv1d–offset)	Linear–offset with gather	0.534 ± 0.120	14.64 ± 3.00	0.618 ± 0.242	7.9	2.16 ± 1.12	5.79 ± 3.88
Abl−2 (no HPO)	Fixed default hyperparameters	0.679 ± 0.213	18.92 ± 5.94	0.871 ± 0.424	51.9	4.06 ± 0.93	17.24 ± 7.80
Abl−3 (no weighting)	Uniform sample weights	0.507 ± 0.101	13.99 ± 2.75	0.555 ± 0.197	−3.1	2.19 ± 1.08	6.06 ± 3.87
Abl−4 (MSE loss)	MSE replaces Huber loss	0.516 ± 0.118	14.15 ± 2.96	0.596 ± 0.242	4	2.42 ± 1.27	7.29 ± 4.96
Abl−5 (meteo–only)	6 meteorological features only	0.515 ± 0.100	14.11 ± 2.64	0.597 ± 0.203	4.2	2.31 ± 0.71	6.06 ± 2.96
Abl−6 (search–only)	5 search features only	0.559 ± 0.155	15.37 ± 4.02	0.667 ± 0.321	16.4	2.80 ± 1.85	10.58 ± 11.40

ΔMSE (%) is the percentage change in MSE relative to the full model. Abl-2 (no HPO) is the most impactful component (+51.9%), driven primarily by degradation at the 7-day horizon (MSE 1.153 vs. 0.394 for Full). Abl-3 (no weighting) shows a negative ΔMSE (−3.1%) because sample weighting prioritizes peak-period accuracy (peak MAE 2.09 vs. 2.19) at a small cost to overall MSE on common target weeks, which are dominated by non-epidemic periods; the weighting is retained because peak-period accuracy is operationally more important in epidemic surveillance.

For each forecast horizon, a separate DeformTime model was trained with output length H equal to the horizon (in weeks; H = 1, 2, 3, 4 for 7, 14, 21, 28 days); horizon-specific evaluation extracts only the final output step (index j = H – 1, corresponding to the exact lead time being evaluated), rather than averaging predictions across different lead times for the same target week. This ensures that the metrics for each horizon reflect only predictions made at that specific lead time.

To assess whether differences in forecast accuracy between models are statistically significant, we performed the Diebold-Mariano (DM) test with Newey-West HAC standard errors to account for serial correlation in multi-step forecast errors. Pairwise DM tests were conducted for each forecast horizon (7, 14, 21, 28 days) using MSE as the loss function. Results are reported as DM statistics with significance levels (^*^p < 0.05, ^**^p < 0.01).

## 3 Results

### 3.1 Seasonal distribution and interannual heterogeneity of ILI% in Beijing

**Figure 2:**
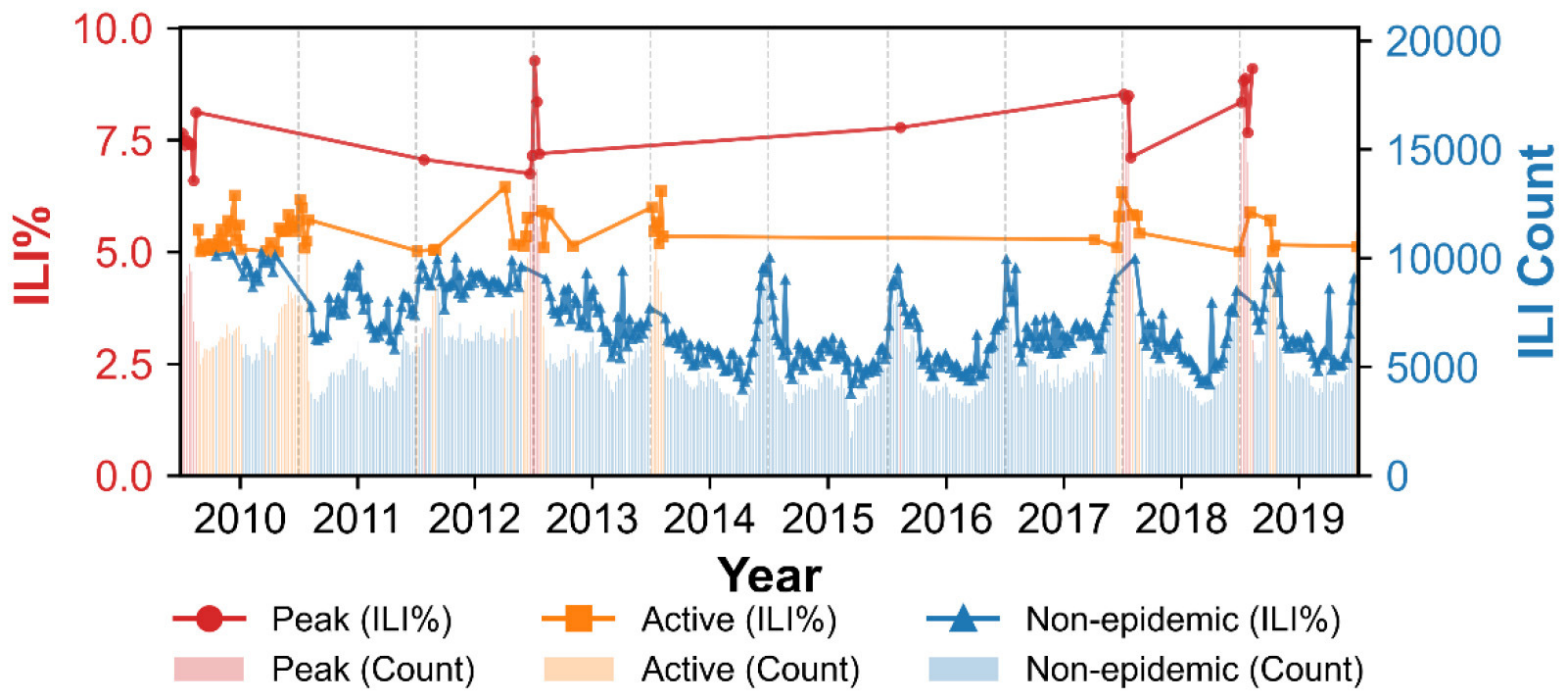
Weekly ILI% and ILI case counts by epidemic activity level in Beijing, 2010–2019.

Figure 2 presents the weekly time series of ILI% and ILI case counts in Beijing from 2010 to 2019, color-coded by epidemic activity level. ILI% exhibits a pronounced unimodal winter–spring seasonality, with epidemic peaks consistently occurring between November and March. However, marked interannual variability in peak intensity, duration, and timing is evident, posing a substantial challenge to model generalization.

Across the study period, years with true peak episodes (ILI% ≥ 6.5%) include 2010, 2012, 2013, 2016, 2018, and 2019, whereas 2011, 2014, 2015, and 2017 did not exhibit clearly defined peak-level weeks (Table 8). The most severe epidemics occurred in 2013 and 2019, with maximum weekly ILI% values of 9.27% and 9.10% respectively, far exceeding the decade average. This intermittent, high-spike pattern characterized by prolonged low-level activity punctuated by abrupt, intense outbreaks, is consistent with the combined effects of cold-dry meteorological conditions that favor influenza virus survival, and population-level behavioral responses amplified by intensive online information-seeking.

**Table 8:** Descriptive statistics of ILI% by year and activity level, Beijing 2010–2019.

Year	Mean	SD	Max	Min	Non–epidemic (mean)	Active (mean)	Peak (mean)
2010	5.45	0.9	8.12	4.22	4.68	5.36	7.44
2011	3.83	0.75	6.17	2.84	3.63	5.64	——
2012	4.67	0.74	7.15	3.73	4.36	5.38	6.99
2013	3.97	1.3	9.27	2.64	3.54	5.5	8.28
2014	3.21	1.08	6.37	1.95	2.89	5.67	——
2015	2.74	0.53	4.9	1.86	2.74	——	——
2016	3.01	0.94	7.78	2.13	2.92	——	7.78
2017	3.5	0.8	6.34	2.58	3.32	5.63	——
2018	3.52	1.62	8.52	2.07	2.92	5.52	8.13
2019	3.89	1.78	9.1	2.37	3.15	5.38	8.56
Overall	3.78	1.35	9.27	1.86	3.42	5.51	7.86

Descriptive statistics by activity level (Table 8) confirm pronounced distributional heterogeneity: non-epidemic weeks maintained a mean ILI% of 3.42% (SD not shown), active weeks averaged 5.51%, and peak weeks averaged 7.86%, with a right-skewed, heavy-tailed distribution (kurtosis > 2). This statistical structure with 82.9% of observations at baseline and only 4.5% at peak level creates an inherently imbalanced training signal, explaining why autoregressive models (e.g., SARIMA) systematically underestimate epidemic peaks and underscoring the need for deep learning architectures capable of exploiting nonlinear exogenous co-variates.

### 3.2 CCF analysis

From 342 candidate variables, 296 passed stationarity testing and entered CCF analysis. After two-stage BH-FDR correction and morphology quality control, 11 predictive features were ultimately retained (Table 9): 6 meteorological variables, 2 PCA composite search indices, and 3 standalone search keywords. All features were nominally significant under exploratory screening (*q* from 10^−3^ to 10^−20^), and 8 of 9 standalone features (88.9%) remained significant under both differenced and raw-series CCF, consistent with the associations not being artifacts of shared seasonality.

**Table 9:** CCF–selected predictive features and seasonality sensitivity.

Categories	Variable	Best lag (wk)^a^	*r* (differenced)^b^	*q* (FDR)^c^	*r* (raw)^d^	Quality	Var. explained^e^
Meteorological	Weekly Max Temperature/周最高气温	3	−0.296	1.9 × 10^−6^	−0.421	Monotone	—
	Weekly Min Temperature/周最低气温	3	−0.259	4.3 × 10^−5^	−0.383	Monotone	—
	Weekly Mean Wind Speed/周平均风速	0	0.205	1.6 × 10^−3^	+0.202^f^	Monotone	—
	Weekly Min Pressure/周最低气压	0	−0.202	1.8 × 10^−3^	0.277	Monotone	—
	Weekly Max Wind Speed/周最大风速	0	0.198	2.3 × 10^−3^	0.232	Monotone	—
	Weekly Extreme Wind Speed/周极大风速	0	0.194	2.9 × 10^−3^	0.135	Good	—
Search Composite Indices (PCA)	Treatment Index	0	−0.450^g^	—	−0.347^g^	Good	82.70%
Symptom Index	0	−0.382^g^	—	−0.420^g^	Good	74.80%
Standalone Search Keywords	Hospital Registration/挂号	0	−0.525	4.3 × 10^−20^	−0.634	Monotone	—
	Community Hospital/社区医院	0	−0.492	2.5 × 10^−17^	−0.678	Good	—
	Immune Enhancement/增强免疫力	0	−0.352	6.5 × 10^−9^	−0.676	Monotone	—

^a^Best lag with maximum |CCF| among FDR–significant lags. Positive lag = variable leads ILI%; 0 = contemporaneous.^b^ |CCF| after seasonal differencing (lag = 52) and first-order differencing where needed (ADF, α = 0.05).^c^ Two-stage BH-FDR q-value (Stage 1: per-variable optimal-lag p-value; Stage 2: BH-FDR across *n* = 296 variables). All q < 0.05.^d^ |CCF| from raw (non-differenced) series, for seasonality sensitivity. ^f^ Weekly Mean Wind Speed had best lag = −4 in the raw series (nowcasting only) but lag = 0 after differencing.^eg^ PCA indices: variance explained by PC1 on original series (^e^); r = Pearson correlation between PC1 and ILI% (^g^), not single-variable CCF. PC1 loadings, Treatment: 牛黄解毒片, 止咳糖浆, 银黄颗粒; Symptom: 胸闷, 口苦, 头晕.

Selected from 296 stationary variables (of 342 candidates). CCF used 2010–2016 data only (Fold 1 training set) to prevent leakage. 8/9 standalone features (88.9%) were also selected under raw-series CCF.

Weekly maximum (*r* = −0.296) and minimum (*r* = −0.259) temperature led ILI% by 3 weeks, with negative correlations indicating that lower temperatures precede higher influenza activity, consistent with the established mechanism that cold conditions facilitate viral survival and indoor crowding. This three-week lead provides a meaningful early-warning window. Wind speed and atmospheric pressure showed contemporaneous associations (lag = 0), capturing the environmental context of active transmission without providing lead time.

All five search-derived features exhibited contemporaneous correlations (lag = 0). The standalone keywords Hospital Registration (*r* = −0.525), Community Hospital (*r* = −0.492), and Immune Enhancement (*r* = −0.352) showed the strongest associations. The two PCA composite indices (Treatment Index: *r* = −0.450, 82.7% variance explained; Symptom Index: *r* = −0.382, 74.8%) captured broader semantic dimensions of treatment-seeking and symptom-awareness behavior. The negative correlations likely reflect that these search terms represent proactive health-seeking behavior that peaks early in the epidemic cycle; as ILI% rises and affected individuals shift from online searching to clinical visits, search volume plateaus, producing a negative contemporaneous association. Critically, these relationships persisted after seasonal differencing (e.g., 挂号: *r* = −0.525 differenced vs. −0.634 raw), confirming independent predictive information beyond shared seasonality.

The 11 features form a temporally layered input set, temperature provides a 3-week lead, while search indices capture the current epidemic trajectory. This combination suits multi-horizon forecasting. In the following section, eight models (Adapted DeformTime, DeformTime-Base, LSTM, XGBoost, Random Forest, SARIMA, ARIMAX, SARIMAX) and three naïve benchmarks (Persistence, Seasonal-naïve, Calendar-based) are evaluated under three-fold rolling-origin cross-validation at 7–28 day horizons, with six ablation experiments further isolating the contributions of model components and feature subsets (meteorological-only, search-only).

### 3.3 Model prediction results

#### 3.3.1 Overall forecasting performance

Table 4 summarizes the forecasting performance of all eleven models across four horizons (7, 14, 21, and 28 days). All metrics are computed on common target weeks, the intersection of target weeks available across all model families for each horizon (*n* = 147, 144, 141, and 138 at 7, 14, 21, and 28 days, respectively), ensuring that all models are evaluated on identical sets of target weeks. Pairwise statistical significance is assessed via the Diebold–Mariano test on aligned weeks (Section 3.3.2).

DeformTime-Ad achieved the lowest MAE at the 7-day and 14-day horizons and the lowest MSE at 7–21 days. At the 7-day horizon, DeformTime-Ad yielded an MAE of 0.392 and MSE of 0.394, representing reductions of 26.2% in MAE and 29.3% in MSE relative to DeformTime-Base (MAE 0.531, MSE 0.557), and 17.3% in MAE and 26.9% in MSE relative to SARIMA (MAE 0.474, MSE 0.539). The advantage was most pronounced at 14 days, where DeformTime-Ad's MAE (0.451) was 19.5% lower than DeformTime-Base (0.560) and 14.7% lower than SARIMA (0.529). At 21 days, DeformTime-Ad attained the lowest MSE (0.699), although SARIMA achieved a marginally lower MAE (0.543 vs. 0.561). At 28 days, DeformTime-Ad's MSE (0.732) was no longer the lowest, as Calendar (0.637), LSTM (0.647), ARIMAX (0.682), SARIMA (0.687), DeformTime-Base (0.727), all attained lower MSE; SARIMA also achieved the lowest MAE (0.600 vs. 0.611 for DeformTime-Ad). This reflects a higher-variance error profile for DeformTime-Ad at longer lead times, where the deformable-attention architecture's advantage in capturing sharp peak transitions is offset by reduced precision during stable periods. Nevertheless, DeformTime-Ad achieved a 61.3% MSE reduction relative to the reporting-lag-corrected persistence benchmark (MSE 1.891) at 28 days. Native-window metrics are provided in Supplementary Table S10.

Among the comparator models, SARIMA and DeformTime-Base were the strongest baselines. SARIMA benefited from its explicit seasonal component (lag-52) and became increasingly competitive at longer horizons, achieving the lowest MAE at 21 and 28 days. DeformTime-Base, sharing the same transformer architecture as DeformTime-Ad but without the proposed adaptations, was consistently outperformed on MAE at all horizons and on MSE at 7–21 days by DeformTime-Ad at all horizons on both MAE and MSE. The three naïve benchmarks, persistence, seasonal-naïve, and calendar-based Fourier regression, were least accurate overall, although persistence remained the strongest short-horizon naïve baseline (7d MAE 0.555) owing to the high week-to-week autocorrelation of ILI%. Notably, ARIMAX and SARIMAX, which incorporated the CCF-selected exogenous variables within a statistical framework, did not improve upon SARIMA; this indicates that the value of multimodal features is better captured by flexible nonlinear models than by linear exogenous-term extensions.

LSTM, XGBoost, and Random Forest showed variable performance: Random Forest was competitive at 7 days (MAE 0.501) but deteriorated markedly at 28 days (MAE 0.745), whereas LSTM exhibited the opposite pattern, improving at longer horizons (28d MAE 0.645, MSE 0.647). This instability across horizons contrasts with the consistent performance of DeformTime-Ad, which maintained the lowest sMAPE at 7, 14, and 28 days and the lowest MSE at 7–21 days.

**Figure 3:**
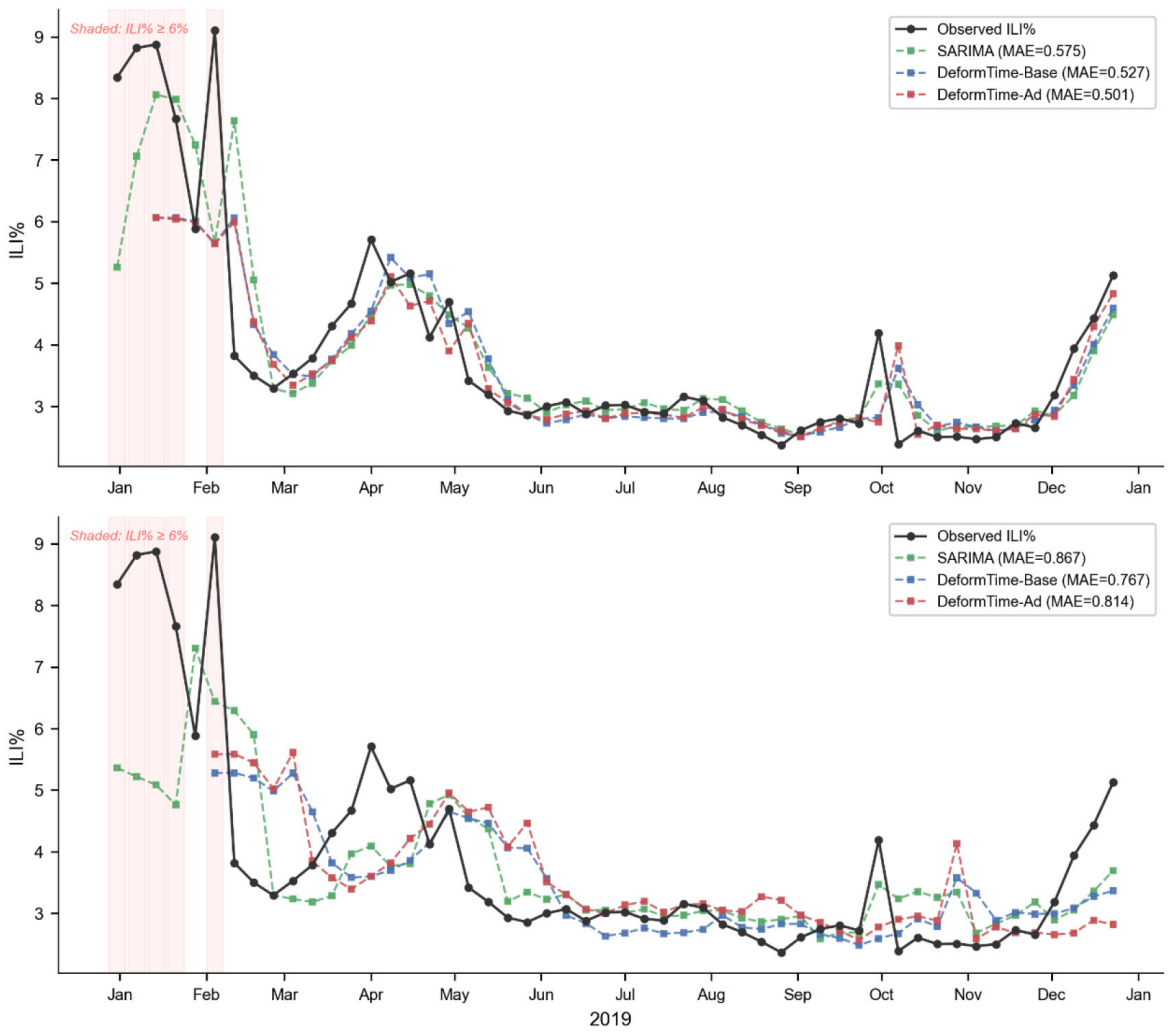
Observed vs. predicted ILI% time series for 2019 (Fold 3) at the 7-day(up) and 28-day(down) horizons.

Figure 3 illustrates the observed and predicted ILI% trajectories for the 2019 test year (Fold 3) at the 7-day and 28-day horizons. At 7 days, all three models tracked the winter epidemic peak (weeks 1–8, ILI% ≥ 6%) with reasonable fidelity; however, SARIMA and DeformTime-Base systematically underestimated peak amplitude, whereas DeformTime-Ad captured both the peak height and the subsequent decline more closely. At 28 days, horizon-induced degradation became visually apparent: all models exhibited temporal lag and amplitude attenuation, yet DeformTime-Ad maintained closer alignment with the observed trajectory during the peak period, while SARIMA produced a smoothed, delayed peak that missed the outbreak timing. These visual patterns corroborate the aggregate metrics in Table 4 and motivate the stratified analysis of peak-period accuracy presented in Section 3.3.3.

#### 3.3.2 Pairwise statistical comparison (diebold–mariano test)

To establish whether the observed performance differences were statistically significant, we conducted pairwise Diebold–Mariano tests with Newey–West HAC standard errors on the aligned squared-error series (Table 5). All comparisons were computed on common target weeks (*n* = 147, 144, 141, and 138 at 7, 14, 21, and 28 days, respectively), ensuring that each pairwise test used identical target weeks for both models.

DeformTime-Ad significantly outperformed the majority of comparator models at shorter horizon, with the advantage narrowing at longer lead times. At 7 days, it was significantly superior to 8 of 10 comparators (*p* < 0.05), including persistence (*p* = 0.029), seasonal-naïve (*p* = 0.001), calendar (*p* < 0.001), ARIMAX (*p* = 0.003), SARIMAX (*p* = 0.006), XGBoost (*p* = 0.022), LSTM (*p* = 0.007), and DeformTime-Base (*p* = 0.022); the comparisons with SARIMA (*p* = 0.113), and Random Forest (*p* = 0.102) were directionally favorable but did not reach significance. At 14 days, DeformTime-Ad was significantly better than 6 of 10 models, including persistence (*p* = 0.014), seasonal-naïve (*p* = 0.002), calendar (*p* = 0.010), XGBoost (*p* = 0.019), Random Forest (*p* = 0.018), and LSTM (*p* = 0.014); the comparison with DeformTime-Base approached but did not reach significance (*p* = 0.065). At 21 days, DeformTime-Ad significantly outperformed only 2 models, persistence (*p* = 0.038) and seasonal-naïve (*p* = 0.045), while the remaining 8 comparisons were non-significant, reflecting the convergence of model performance at longer lead times. At 28 days, DeformTime-Ad significantly surpassed only persistence (*p* = 0.041); all other comparisons, including DeformTime-Base (*p* = 0.951), were non-significant. Notably, DeformTime-Ad was never significantly outperformed by any model at any horizon (0 losses).

The decreasing number of significant wins is consistent with the MSE convergence observed in Table 4 and reflects the diminishing differentiation among models at longer lead times. DeformTime-Base also significantly outperformed most naïve benchmarks at shorter horizons (7d: 3 wins; 14d: 4 wins), confirming that the core deformable attention architecture itself provides a strong predictive foundation. The additional gains from the proposed adaptations, T-DAB, per-fold Optuna tuning, epidemic-level sample weighting, and Huber loss, are quantified in the ablation analysis (Section 3.3.4).

#### 3.3.3 Stratified Evaluation by Epidemic Intensity

Because accurate prediction during epidemic peaks is of greatest public health importance, we stratified predictions into three levels using data-driven thresholds derived from each fold's training set, peak (ILI% ≥ mean + 2 SD), active (mean + 1 SD ≤ ILI% < mean + 2 SD), and non-epidemic (ILI% < mean + 1 SD). Table 6 reports the stratified metrics averaged across four horizons on common target weeks, ensuring that all models are evaluated on the same set of epidemic weeks.

At the peak level (*n* = 5 common peak weeks, drawn from Fold 2 and Fold 3 only), Persistence achieved the lowest MAE (0.78) and MSE (0.88), reflecting the high autocorrelation of ILI% during the sustained plateau characteristic of the included peak weeks, where the previous observation is a good proxy. However, this advantage would not generalize to the rising and falling flanks of epidemic curves, which are excluded from the common-week peak sample. Among the remaining models, SARIMA ranked second (MAE 1.83, sMAPE 23.78%), benefiting from its explicit seasonal component, followed by Random Forest (2.08) and DeformTime-Ad (2.09, sMAPE 27.78%). The advantage of DeformTime-Ad over DeformTime-Base was substantial: 31.9% lower MAE (2.09 vs. 3.07), 44.6% lower MSE (5.49 vs. 9.92), and 37.4% lower sMAPE (27.78% vs. 44.37%). LSTM exhibited the worst peak accuracy (MAE 4.42, MSE 20.39, sMAPE 70.83%), suggesting that standard recurrent architectures struggle to capture the nonlinear dynamics of epidemic surges.

At the active level (*n* = 36), DeformTime-Ad achieved the lowest MAE (1.03), sMAPE (21.39%), and MSE (1.62), followed by XGBoost (MAE 1.13) and Random Forest (1.15). In the non-epidemic stratum (*n* = 529), DeformTime-Ad again led with the lowest MAE (0.45) and sMAPE (13.02%), though all models performed comparably (MAE 0.45–0.66), indicating that the principal benefit of the proposed model, and of the multimodal feature set, lies in capturing epidemic dynamics rather than in tracking baseline ILI fluctuations.

These results demonstrate that the epidemic-level sample weighting scheme (peak weight 3.0, active 2.0, non-epidemic 1.0) successfully biased the model toward accurate peak prediction, DeformTime-Ad's peak MAE was 31.9% lower than that of the unadapted DeformTime-Base, while maintaining the lowest MAE in the active and non-epidemic strata. The very limited peak sample (*n* = 5; 0 at the 28-day horizon) constrains the statistical reliability of the peak-period ranking; native-window stratified results with a larger peak sample are provided in Supplementary Table S11.

**Figure 4:**
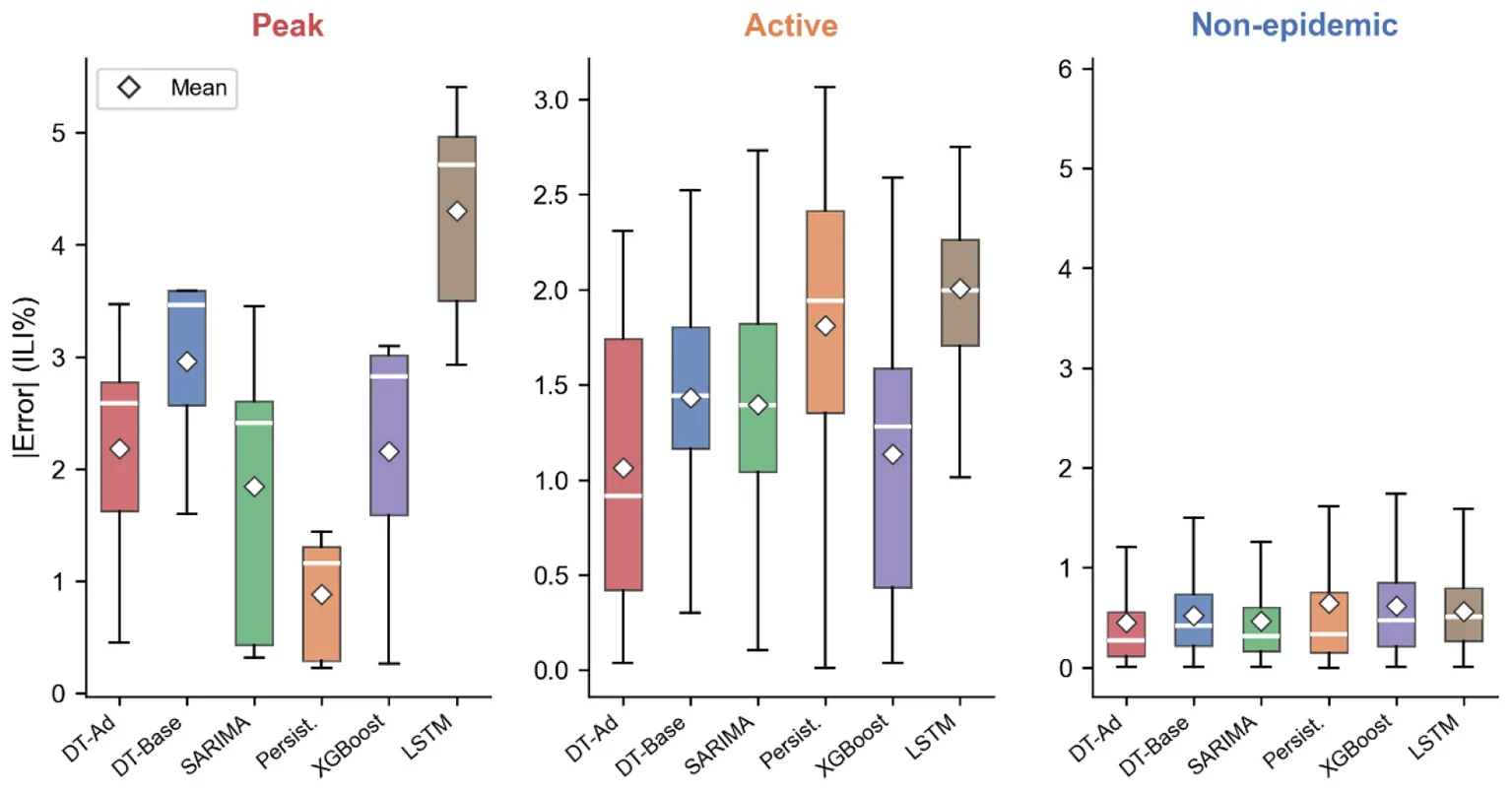
Prediction error distribution by epidemic intensity.

Figure 4 complements the tabulated mean ± SD values (Table 6) by revealing the full error distribution across epidemic strata for six representative models. During peak periods, Persistence exhibited the lowest errors, consistent with its strong peak MAE (0.78), though with wide variance (SD 0.60) reflecting the heterogeneous nature of the five common peak weeks. DeformTime-Ad showed a substantially lower median and narrower spread than DeformTime-Base, visually confirming the 31.9% peak MAE reduction. LSTM displayed the highest median with a compact spread, indicating uniformly poor peak accuracy rather than occasional failures. In the active stratum, DeformTime-Ad exhibited the lowest median error and narrowest interquartile range, while Persistence and LSTM showed the highest errors. In the non-epidemic stratum, all six models converged to similarly narrow, low-error distributions, visually confirming that model differentiation is concentrated at epidemic peaks and active periods, precisely where public health utility is greatest.

#### 3.3.4 Ablation studies

To isolate the contribution of each component, six ablation variants were evaluated under the identical rolling-origin protocol on common target weeks (Table 7). Results are averaged across the four horizons.

Per-fold hyperparameter optimization was the single most impactful adaptation, removing it (Abl-2) increased MSE by 51.9% (0.871 vs. 0.573), driven primarily by severe degradation at the 7-day horizon (MSE 1.153 vs. 0.394). The effect was especially pronounced during epidemic peaks, where peak MAE rose from 2.09 to 4.06 and peak MSE from 5.49 to 17.24, underscoring the critical role of tuned learning dynamics in short-horizon and peak-period forecasting.

The task-decoupled attention block (Abl-1, Conv1d-offset replaced with linear-offset) ranked second among architectural components (+7.9% MSE), confirming that deformable temporal attention captures non-stationary cross-variable dependencies that fixed-kernel architectures miss. Replacing Huber loss with standard MSE loss (Abl-4) increased overall MSE by 4.0% and peak MSE by 32.8% (7.29 vs. 5.49), confirming Huber's role in suppressing large epidemic-peak errors without inflating typical-week bias.

Epidemic-level sample weighting (Abl-3) exhibited a nuanced trade-off: it improved peak-period MAE (2.09 vs. 2.19 without weighting) and peak MSE (5.49 vs. 6.06) at a small cost to overall MSE (−3.1%), because common target weeks are dominated by non-epidemic periods where uniform weighting is marginally more efficient. The weighting is retained because peak-period accuracy is the operationally critical objective in epidemic early warning.

Feature subset ablations (Abl-5, Abl-6) confirmed that both modalities contribute complementary signals; their horizon-dependent contributions are analyzed in Section 3.3.5.

#### 3.3.5 Feature subset analysis

Feature importance exhibited a marked horizon dependence (Supplementary Table S13). At the 7-day horizon, the search-only model (Abl-6; MSE 0.428) outperformed the meteorology-only model (Abl-5; MSE 0.461), indicating that Baidu search indices carry the primary short-term predictive signal, consistent with their stronger CCF correlations (|*r*| = 0.35–0.53) and contemporaneous association with ILI%. At longer horizons (14–28 days), the pattern reversed: the meteorology-only model (e.g., 28d MSE 0.685) substantially outperformed the search-only model (28d MSE 0.734), reflecting the lead-time advantage of meteorological variables (temperature lag = 3 weeks). Averaged across all four horizons, Abl-5 (meteo-only) yielded MSE 0.597 (+4.2%) and Abl-6 (search-only) yielded MSE 0.667 (+16.4%), confirming that both feature modalities contribute complementary, horizon-dependent signals.

This complementarity explains why the full multimodal model outperforms either single-modality variant: search behavior captures immediate public health awareness that drives short-horizon accuracy, while meteorological variables provide longer-lead environmental drivers that sustain performance at 14–28 days. Although the meteorology-only model achieved marginally lower MSE at the longest horizons (21d: 0.698 vs. 0.699; 28d: 0.685 vs. 0.732), the full model's substantial advantage at 7–14 days, where forecasting is most actionable for outbreak response, yields the best 4-horizon average (MSE 0.573) and the strongest peak-period accuracy among ablation variants (peak MAE 2.09 vs. 2.31 for Abl-5 and 2.80 for Abl-6). Multimodal inputs further improve robustness to search-index volatility and seasonal meteorological anomalies, ensuring stable performance across the diverse epidemic patterns encountered in the three test years.

## 4 Discussion

### 4.1 Principal findings

This study developed an Adapted DeformTime model for weekly ILI% forecasting in Beijing, China, integrating CCF-selected meteorological and Baidu search-index features within a deformable-attention transformer framework enhanced by task-decoupled attention, epidemic-level sample weighting, per-fold hyperparameter optimization, and Huber loss. Under a rigorous rolling-origin protocol (3 folds × 4 horizons, all evaluation on held-out test years 2017–2019), DeformTime-Ad achieved the lowest MAE at 7–14 days (0.39–0.45) and the lowest MSE at 7–21 days. In Diebold–Mariano tests, it was significantly superior to 8, 6, 2, and 1 of 10 comparators at 7, 14, 21, and 28 days, respectively, with zero significant losses at any horizon. The model's advantage over DeformTime-Base was most consequential during epidemic peaks, where MAE was reduced by 31.9%.

### 4.2 Contribution of model components

Per-fold hyperparameter optimization was the most impactful single adaptation (MSE +51.9% when removed), driven primarily by severe degradation at the 7-day horizon (MSE 1.153 vs. 0.394 for the Full model), underscoring the critical role of tuned learning dynamics in short-horizon forecasting. The task-decoupled attention block (Conv1d-offset) ranked second (+7.9%), confirming that deformable temporal attention captures non-stationary cross-variable dependencies that fixed-kernel architectures miss. Epidemic-level sample weighting exhibited a nuanced trade-off: it improved peak-period MAE (2.09 vs. 2.19 without weighting) at a small cost to overall MSE (−3.1%), because common target weeks are dominated by non-epidemic periods where uniform weighting is marginally more efficient. The weighting is retained because peak-period accuracy is the operationally critical objective in epidemic early warning. The Huber-loss ablation (+4.0%) confirmed its role in suppressing large epidemic errors without inflating typical-week bias.

### 4.3 Role of meteorological and search features

Feature importance exhibited a clear horizon dependence. At the 7-day horizon, Baidu search indices carried the primary predictive signal (search-only MSE 0.428 vs. meteorology-only MSE 0.461), reflecting the public's anticipatory health-seeking behavior and its contemporaneous association with ILI%. At longer horizons (14–28 days), meteorological variables became dominant (e.g., 28d: meteo-only MSE 0.685 vs. search-only MSE 0.734), reflecting the lead-time advantage of temperature (lag ≈ 3 weeks) as an environmental driver of viral transmission. This complementarity, search behavior for short-term awareness, meteorology for longer-lead environmental forcing, motivates the multimodal design. The observation that ARIMAX and SARIMAX did not improve upon univariate SARIMA underscores that the predictive value of multimodal features is contingent on the model's capacity to exploit nonlinear and interactive effects.

### 4.4 Performance across forecast horizons

DeformTime-Ad maintained the lowest MAE at 7–14 days and the lowest MSE at 7–21 days, though its advantage narrowed at longer lead times. At 7 days, DeformTime-Ad was dominant (MAE 0.392, MSE 0.394; 8 of 10 DM wins). As the horizon extended, the reporting-lag-corrected persistence benchmark eroded rapidly (MSE rising from 0.844 to 1.891), while DeformTime-Ad degraded more gracefully in MAE (0.392 to 0.611). At 21 and 28 days, SARIMA achieved a marginally lower MAE (0.543 vs. 0.561 and 0.600 vs. 0.611), benefiting from its explicit seasonal component at longer lead times. At 28 days, DeformTime-Ad significantly outperformed persistence in the DM test (*p* = 0.041), achieving a 61.3% MSE reduction; however, its MSE (0.732) was no longer the lowest, as Calendar (0.637), LSTM (0.647), ARIMAX (0.682), SARIMA (0.687), DeformTime-Base (0.727) all attained lower MSE at this horizon. This reflects a higher-variance error profile at the longest lead time, where the model's sensitivity to peak transitions incurs larger squared errors during stable periods. The decreasing number of DM wins is consistent with this convergence.

### 4.5 Epidemic peak prediction

On common target weeks, only 5 peak-week predictions were available per model (3 at 7d, 1 at 14d, 1 at 21d, 0 at 28d), limiting the statistical reliability of the peak-period ranking. Within this constraint, DeformTime-Ad attained a peak MAE of 2.09, 31.9% lower than DeformTime-Base (3.07) and 44.6% lower peak MSE (5.49 vs. 9.92), and the lowest active-period MAE (1.03) and non-epidemic MAE (0.45) among all models. Persistence exhibited strong peak-period performance (MAE 0.78) on the common peak weeks, reflecting the sustained plateau characteristic of the included peak weeks where the previous observation is a good proxy; this advantage would not generalize to the rising and falling flanks of epidemic curves, which are excluded from the common-week peak sample. LSTM exhibited the worst peak accuracy (MAE 4.42, MSE 20.39). All models performed similarly during non-epidemic periods (MAE 0.45–0.66), meaning model differentiation is concentrated at epidemic peaks and active periods. The very small peak sample (*n* = 5) is an acknowledged limitation; native-window stratified results with a larger peak sample are provided in Supplementary Table S11.

### 4.6 Comparison with existing studies

Compared with prior ILI forecasting studies that predominantly relied on single-modality inputs, either univariate surveillance time series (17, 23, 36) or search-index data alone (18, 37), the present work suggests that principled multimodal fusion within a deformable-attention architecture yields accuracy gains, particularly at 1–3 week horizons. While Santillana et al. (19) and Wang et al. (20) similarly integrated heterogeneous data sources, neither employed formal statistical feature selection with multiple-comparison correction; our two-stage CCF framework with Benjamini–Hochberg FDR control and seasonality sensitivity analysis provides a transparent, reproducible pipeline, though the CCF analysis serves as an exploratory screening procedure rather than a confirmatory inference framework. Furthermore, the three-fold rolling-origin protocol with strict per-fold leakage prevention, CCF computation confined to the Fold 1 training period (2010–2016) and frozen across all folds, addresses a pervasive weakness in prior epidemiological forecasting studies (32, 36).

### 4.7 Limitations

Several limitations warrant acknowledgment. First, the study used single city (Beijing) data, and one search engine (Baidu); generalizability to other populations and platforms requires prospective, multi-city external validation. Second, the rolling-origin protocol covers only three test years (2017–2019), with the COVID-19 period excluded due to profound anomalies in ILI reporting and search behavior. Third, common-target-week evaluation, while ensuring fair comparison, reduces the peak-week sample to only 5 observations (0 at 28 days), limiting the statistical power of the stratified peak analysis; the per-year array segmentation additionally imposes a 2-week burn-in for supervised models. Fourth, at 21–28 days, SARIMA achieved a lower MAE than DeformTime-Ad (0.543 vs. 0.561 and 0.600 vs. 0.611), and at 28 days, Calendar, LSTM, ARIMAX, SARIMA, DeformTime-Base all attained lower MSE, reflecting DeformTime-Ad's higher-variance error profile at long lead times. Fifth, the uniform 2-week lookback window, while ensuring comparability across models, may disadvantage sequence models such as LSTM that benefit from longer input sequences to capture annual seasonality; architecture-specific lookback optimization is a promising direction, though it would require careful design to maintain comparability. Finally, the CCF analysis provides exploratory feature screening with partial multiple-comparison control but does not account for data-driven lag selection or residual serial dependence; prewhitening was not performed, and the fixed feature set selected on 2010–2016 may not fully capture evolving search behavior and meteorological sensitivity in later years.

## 5 Conclusions

This study suggests that an Adapted DeformTime model integrating CCF- screened meteorological and Baidu search-index features improves weekly ILI% forecasting in Beijing, China, particularly at the 1–3 week horizons. DeformTime-Ad achieved the lowest MAE at 7–14 days and the lowest MSE at 7–21 days, and was never significantly outperformed in Diebold–Mariano tests, though its MAE and MSE advantages narrowed at longer horizons. The principal drivers of this improvement are per-fold hyperparameter optimization, the task-decoupled attention block, and epidemic-level sample weighting, the last of which improves peak-period accuracy at a small overall-MSE trade-off. Meteorological and search-index features provide complementary, horizon-dependent signals, with search behavior dominant at short lead times and meteorological variables at longer lead times. These findings support the potential utility of multimodal deep-learning early-warning systems that prioritize peak-period accuracy, pending prospective and multi-city external validation. The framework, combining exploratory CCF-based feature screening, leakage-free rolling-origin evaluation on common target weeks, and component-level ablation, offers a methodology for infectious disease forecasting in data-rich settings.

## Data Availability

Publicly available datasets were analyzed in this study. This data can be found here: The Baidu Search Index data are publicly available at https://index.baidu.com. Meteorological data are accessible via http://data.cma.cn upon registration. ILI surveillance data from Beijing CDC are not publicly available due to ethical restrictions but may be requested from the corresponding authors subject to institutional approval.
