# Supplementary material for: Fusing meteorological factors and baidu search index with an adapted deformtime model to predict influenza-like illness in Beijing
Source: Front Public Health. 2026 Sep 3;14:1856514. doi: 10.3389/fpubh.2026.1856514 (PMC13582498; doi:10.3389/fpubh.2026.1856514)
Supplement: Supplementary file 1 [file Data_Sheet_1.pdf]

Supplementary Material

Fusing meteorological factors and Baidu Search Index with an adapted DeformTime model to predict influenza-like illness in Beijing

Lingling Shen[1], Yajun Xiong[1]*, Zhaobin Sun[2]*
[1] Beijing Meteorological Data Center, Beijing 100089, China
[2] Chinese Academy of Meteorological Sciences, Beijing 100081, China
**Corresponding authors: Yajun Xiong (xyajun78@163.com); Zhaobin Sun (szb850804@163.com)*

Supplementary Appendix

Supplementary Table S0. ILI% activity-level thresholds (based on the sample mean (3.78%) and standard deviation (1.35%) of the full study period), epidemiological meaning, and sample proportions.

Level	Threshold	Epidemiological meaning	Sample proportion (%)
Non-epidemic	ILI% < 5.0%	Baseline transmission level	82.94
Active	5.0% ≤ ILI% < 6.5%	Elevated activity; mean + 1 SD	12.56
Peak	ILI% ≥ 6.5%	High-intensity outbreak; mean + 2 SD	4.5

Note: This table presents the overall descriptive statistics for reference; actual model evaluation uses per-fold training-set thresholds (see Table 2 and Section 2.1)

Supplementary Text S1. CCF-Based Feature Selection and Dimensionality Reduction

S1.1 Data sources and preprocessing

To construct a parsimonious predictive feature set, cross-correlation function (CCF) analysis with lagged association screening was applied to 342 candidate exogenous variables from the 2010–2016 period (365 weekly observations). Candidate variables comprised 20 meteorological variables (temperature, wind speed, atmospheric pressure, humidity, etc.) and 322 Baidu Search Index keywords spanning four semantic categories: symptoms, treatment interventions, healthcare-seeking behavior, and prevention. CCF analysis was restricted to 2010–2016 data (i.e., the Fold 1 training set) to ensure that the feature selection stage did not access any validation-fold test sets (2017–2019), thereby preventing data leakage. The target variable was weekly ILI%.

S1.2 Time series stationarization

Because influenza surveillance data, meteorological measurements, and search indices all exhibit strong seasonality and autocorrelation, direct computation of cross-correlations risks spurious associations. All series entering CCF analysis were therefore stationarized as follows:

Target variable (ILI%): Seasonal differencing (lag = 52 weeks) was applied first, followed by the Augmented Dickey–Fuller (ADF) test for stationarity ($\alpha = 0.05$). If non-stationary, first-order differencing was additionally applied.

Exogenous variables: Each candidate variable underwent the same sequential procedure—seasonal differencing → ADF test → additional first-order differencing if needed—and the differencing order was recorded (diff_order = 1: seasonal differencing only; diff_order = 2: seasonal + first-order differencing). Variables that remained non-stationary after differencing (46/342) were excluded, yielding 296 variables that passed stationarity testing for CCF computation.

Differencing-order alignment: When computing the CCF between a given variable and ILI%, the ILI% differenced series was matched to that variable's diff_order (diff_order = 1 paired with seasonally differenced ILI%; diff_order = 2 paired with seasonally + first-order differenced ILI%), ensuring that both series were cross-correlated at the same differencing order.

S1.3 Cross-correlation function computation

For each stationarized variable, the Pearson cross-correlation coefficient and corresponding p-value were computed against ILI% over lags $\tau = -4$ to $+4$ weeks (9 lag orders). Lag sign convention: $\tau > 0$ indicates that the exogenous variable leads ILI% (X_t correlated with $Y_{t+\tau}$), which is usable for forecasting; $\tau < 0$ indicates that the exogenous variable lags ILI%, which is applicable only to nowcasting.

S1.4 Multiple comparison control

To control the large-scale multiple testing inherent in 342 variables × 9 lags, a two-stage Benjamini–Hochberg false discovery rate (FDR) correction was employed:

Stage 1: For each variable, the p-value corresponding to the lag with the maximum |CCF| was selected as that variable's candidate best-lag p-value.

Stage 2: A single BH-FDR correction was applied across all variables' best-lag p-values (number of tests = number of variables = 296).

This design is preferable to a single FDR correction over all variable × lag combinations, because the 9 lags of the same variable are highly correlated, and per-lag independent correction would be excessively conservative. Variables with $q < 0.05$ after correction were taken to suggest evidence for a potential lagged association with ILI%.

S1.5 CCF morphology quality check and forecast usability

Significance at a single lag alone is insufficient to confirm the reliability of the lag structure. Each variable's CCF morphology was therefore classified into four quality tiers:

good: Isolated peak (best lag within [−3, +3], with at least one adjacent lag non-significant) or ⩽ 3 significant lags.

monotone: ⩾ 6 significant lags (persistently significant, no clear peak).

boundary: Best lag at ±4 boundary (true lag may lie further out).

weak: No significant lags.

Feature inclusion rule: A variable was deemed forecast-usable (forecast_usable = TRUE) if it was FDR-significant AND had best_lag ⩾ 0. Variables with best_lag < 0 were flagged as "Nowcasting Only" and exported for nowcasting discussion but excluded from predictive models. For monotone-tier variables, an additional threshold of |CCF| ⩾ 0.15 was required for inclusion to avoid introducing weak signals.

S1.6 Category-wise PCA for search terms

FDR-significant and forecast-usable search terms were grouped by semantic category (per the keyword dictionary) into four classes: Symptom, Treatment Intervention, Healthcare-seeking, and Prevention. For sufficiently large categories (Symptom: 39 terms; Treatment Intervention: 70 terms), independent principal component analysis (PCA) was performed, and the first principal component (PC1) was retained as the composite search index for that category (symptom_index, treatment_index). For smaller categories (Healthcare-seeking: 5 terms; Prevention: 2–3 terms), PCA was deemed unstable due to insufficient sample size; instead, the top 1–2 keywords by |CCF| within each category were directly selected as standalone features (挂号，社区医院，增强免疫力), preserving their physical interpretability. Meteorological variables were not subjected to PCA and were retained in their original form to maintain meteorological interpretability.

Although PC1 was validated against ILI% on differenced series to establish the de-seasonalized independent correlation, the composite indices used for downstream modeling were generated on the original (non-differenced) series: symptom_index explained 74.8% of variance and correlated with ILI% at r = −0.420 (p < 0.001); treatment_index explained 82.7% of variance and correlated at r = −0.347 (p < 0.001). PC1 loading analysis indicated that symptom_index was primarily driven by respiratory symptom terms (chest tightness, bitter taste, dizziness), while treatment_index was driven by medication terms (Niuhuang Jiedu Pill, cough syrup, Yinhuang Granules), confirming satisfactory semantic validity.

S1.7 Final feature set

Through the above pipeline, 11 predictive features were retained (Supplementary Table S1): 6 meteorological variables (weekly maximum/minimum temperature, weekly mean wind speed, weekly minimum pressure, weekly maximum/extreme wind speed), 2 composite search indices (symptom_index, treatment_index), and 3 standalone search keywords (挂号 [hospital registration], 社区医院 [community hospital], 增强免疫力 [immunity enhancement]).

S1.8 CCF screening results

Of the 296 variables that passed stationarity testing, 122 satisfied both FDR significance and forecast usability (best_lag ≥ 0), comprising 6 meteorological variables and 116 search terms. An additional 137 variables with best_lag < 0 were exported as nowcasting-only signals. Among the selected features, the search keywords 挂号 (r = −0.525, lag = 0) and 社区医院 (r = −0.492, lag = 0) exhibited the strongest contemporaneous correlations with ILI%, while temperature variables (weekly maximum temperature: r = −0.296, lag = 3; weekly minimum temperature: r = −0.259, lag = 3) led ILI% by 3 weeks, consistent with the epidemiological mechanism whereby low temperatures facilitate influenza transmission.

S1.9 Seasonality sensitivity analysis

To verify that the associations identified by CCF were not merely reflections of shared seasonality rather than independent predictive information, a sensitivity analysis was conducted by re-running CCF on the original (non-differenced) series and comparing variable selection with the differenced CCF results. The findings were:

8 of 9 standalone final features (88.9%) were selected under both approaches, confirming robustness to seasonality removal.

3 standalone search terms exhibited strong correlations with ILI% in the raw series (r = −0.63 to −0.68) and remained significant after differencing (r = −0.35 to −0.53), demonstrating that their associations are not solely driven by shared seasonality.

60 additional variables were selected only after differencing, representing signals that emerge only upon removal of the seasonal trend—i.e., variables with de-seasonalized independent associations not visible in the raw series.

These results suggest that the features selected in this study possess independent predictive value beyond shared seasonal patterns.

Supplementary Table S1. Final 11 CCF-selected predictive features

#	Feature	Type	Category	Best Lag (weeks)	Max |CCF|	CCF Quality	Variance Explained (%)	r with ILI%
1	Weekly Max Temperature	Meteorological	—	3	0.296	monotone	—	—
2	Weekly Min Temperature	Meteorological	—	3	0.259	monotone	—	—
3	Weekly Mean Wind Speed	Meteorological	—	0	0.205	monotone	—	—
4	Weekly Min Pressure	Meteorological	—	0	0.202	monotone	—	—
5	Weekly Max Wind Speed	Meteorological	—	0	0.198	monotone	—	—
6	Weekly Extreme Wind Speed	Meteorological	—	0	0.194	good	—	—
7	Treatment Index	Search PCA	Treatment Intervention	0	0.450	good	82.7	−0.347
8	Symptom Index	Search PCA	Symptom	0	0.382	good	74.8	−0.420
9	挂号 (Hospital Registration)	Search Standalone	Healthcare-seeking	0	0.525	monotone	—	−0.525
10	社区医院 (Community Hospital)	Search Standalone	Healthcare-seeking	0	0.492	good	—	−0.492
11	增强免疫力 (Immunity Enhancement)	Search Standalone	Prevention	0	0.352	monotone	—	−0.352

Note: Best lag selected as the lag with maximum |CCF| among FDR-significant lags. Positive lag indicates the exogenous variable leads ILI% (usable for forecasting); 0 indicates contemporaneous association. Max |CCF| computed after seasonal differencing (lag = 52 weeks) and, where necessary, first-order differencing to achieve stationarity (ADF test, α = 0.05). Two-stage Benjamini–Hochberg FDR: Stage 1 selects each variable's optimal-lag p-value; Stage 2 applies BH-FDR across all variables (n = 296). All q < 0.05 . CCF quality: good = isolated peak; monotone = persistently significant across lags. For PCA composite indices, variance explained and r refer to PC1 on original (non-differenced) series. CCF analysis used 2010–2016 data only (Fold 1 training set) to prevent test-set leakage.

Supplementary Table S2. CCF screening summary

Screening Stage	Count	Details
Initial candidate variables	342	20 meteorological + 322 search terms
Failed stationarity (ADF α = 0.05)	46	Excluded after seasonal + first-order differencing
Passed stationarity → entered CCF	296	—
FDR-significant (two-stage BH-FDR, q < 0.05)	268	—
Forecast-usable (FDR-significant AND best lag ≥ 0)	122	6 meteorological + 116 search terms
Nowcasting-only (best_lag < 0)	137	Exported for nowcasting discussion, excluded from models
FDR-significant but excluded (weak/boundary quality)	9	Failed morphology quality check
Final feature set (post-PCA + standalone selection)	11	6 meteorological + 2 PCA indices + 3 standalone search terms

Supplementary Table S3. Seasonality sensitivity analysis: raw vs. differenced CCF comparison

Metric	Raw (non-differenced) CCF	Differenced CCF	Overlap
Forecast-usable variables	102	122	62 (50.8% of differenced)
Standalone final features selected	8/9 (88.9%)	9/9 (100%)	8/9 (88.9%)

Standalone Feature	Raw best_lag	Raw |CCF|	Diff best_lag	Diff |CCF|	Robust?
Weekly Max Temperature	—	—	3	0.296	✓
Weekly Min Temperature	—	—	3	0.259	✓
Weekly Mean Wind Speed	−4	—	0	0.205	✗ (lag flips)
Weekly Min Pressure	—	—	0	0.202	✓
Weekly Max Wind Speed	—	—	0	0.198	✓
Weekly Extreme Wind Speed	—	—	0	0.194	✓
挂号	0	−0.63 ~ −0.68	0	−0.525	✓
社区医院	0	−0.63 ~ −0.68	0	−0.492	✓
增强免疫力	0	−0.63 ~ −0.68	0	−0.352	✓

Note: Raw CCF was computed on original (non-differenced) series; differenced CCF used seasonal (lag = 52) ± first-order differencing per ADF test. "Robust" = selected under both approaches with consistent best_lag ⩾ 0. Weekly Mean Wind Speed had best_lag = −4 in the raw series (nowcasting only) but lag = 0 after differencing. The 3 standalone search terms showed strong correlations in both approaches (raw r = −0.63 to −0.68; differenced r = −0.35 to −0.53), confirming that their associations are not solely driven by shared seasonality.

Supplementary Figure S1

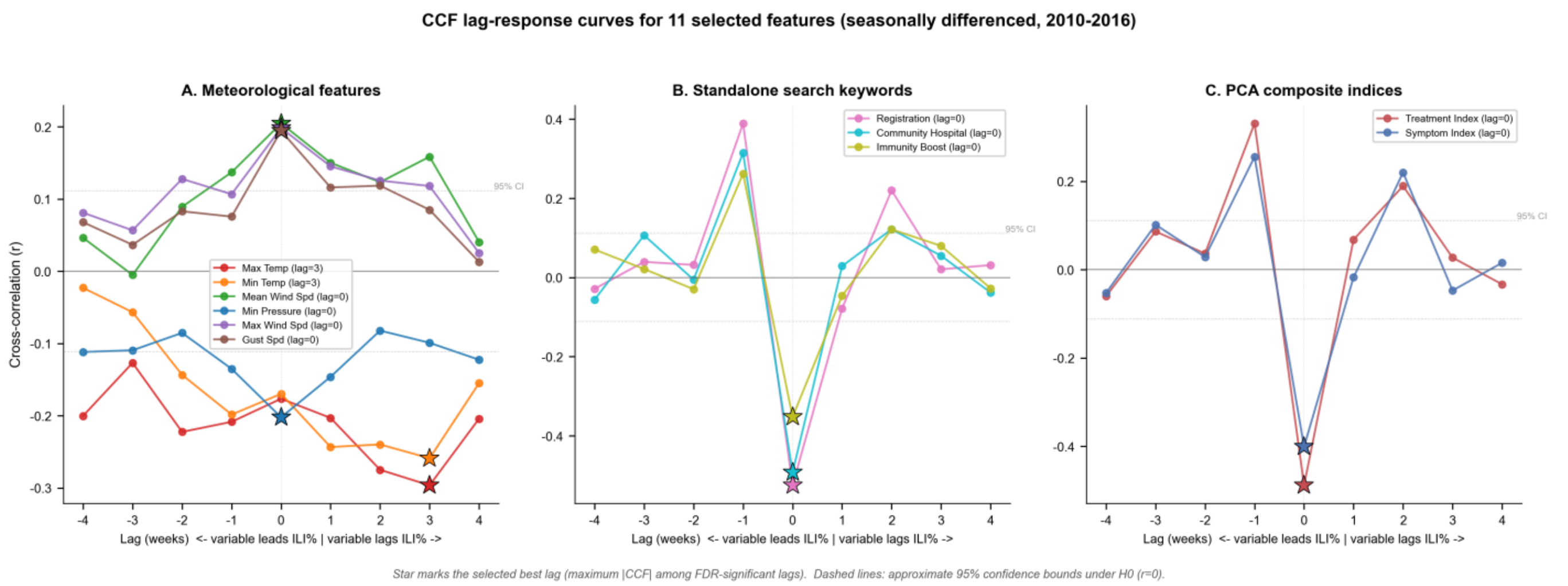

Figure S1 CCF lag-response curves for all 11 selected features, showing the full cross-correlation function over lags −4 to +4 weeks. Star markers (★) indicate the best lag; dashed horizontal lines indicate the 95% confidence interval. Panels are organized by feature type: (A) meteorological variables, (B) standalone search keywords, (C) PCA composite indices.

Supplementary Table S4. Diebold-Mariano Test P-Value Matrices

Pairwise Diebold-Mariano tests with Newey-West HAC standard errors (loss = MSE). Each cell shows the two-sided p-value for H0: equal forecast accuracy between the row and column models. P < 0.05 marked with *. The test is symmetric: p(A,B) = p(B,A).

Abbreviations: Persist. = Persistence; RandForest = Random Forest; DT-Base = DeformTime-Base; DT-Ad = Adapted DeformTime (proposed).

Panel A: 7-day horizon (n = 147 aligned weeks)

Row \ Col	DT-Ad	DT-Base	SeasonalNaive	Persist.	RandForest	Calendar	SARIMAX	ARIMAX	SARIMA	XGBoost	LSTM
**DT-Ad**	**—**	**0.021***	**0.001***	**0.029***	**0.102**	**<0.001***	**0.006***	**0.003***	**0.113**	**0.021***	**0.007***
**DT-Base**	**0.021***	**—**	**0.002***	**0.158**	**0.827**	**0.018***	**0.577**	**0.761**	**0.857**	**0.558**	**0.012***
**SeasonalNaive**	**0.001***	**0.002***	**—**	**0.16**	**0.004***	**0.066**	**0.011***	**0.011***	**0.004***	**0.009***	**0.1**
**Persist.**	**0.029***	**0.158**	**0.16**	**—**	**0.066**	**0.957**	**0.182**	**0.183**	**0.064**	**0.196**	**0.749**
**RandForest**	**0.102**	**0.827**	**0.004***	**0.066**	**—**	**0.029***	**0.449**	**0.597**	**0.984**	**0.138**	**0.066**
**Calendar**	**<0.001***	**0.018***	**0.066**	**0.957**	**0.029***	**—**	**0.059**	**0.037***	**0.020***	**0.068**	**0.633**

**SARIMAX**	0.006*	0.577	0.011*	0.182	0.449	0.059	—	0.499	0.419	0.885	0.081
**ARIMAX**	0.003*	0.761	0.011*	0.183	0.597	0.037*	0.499	—	0.659	0.665	0.073
**SARIMA**	0.113	0.857	0.004*	0.064	0.984	0.020*	0.419	0.659	—	0.457	0.049*
**XGBoost**	0.021*	0.558	0.009*	0.196	0.138	0.068	0.885	0.665	0.457	—	0.113
**LSTM**	0.007*	0.012*	0.1	0.749	0.066	0.633	0.081	0.073	0.049*	0.113	—

Panel B: 14-day horizon (n = 144 aligned weeks)

Row \ Col	DT-Ad	DT-Base	SeasonalNaive	Persist.	RandForest	Calendar	SARIMAX	ARIMAX	SARIMA	XGBoost	LSTM
**DT-Ad**	—	0.065	0.002*	0.014*	0.018*	0.010*	0.052	0.056	0.2	0.019*	0.014*
**DT-Base**	0.065	—	0.004*	0.028*	0.073	0.046*	0.155	0.247	0.504	0.121	0.025*
**SeasonalNaive**	0.002*	0.004*	—	0.86	0.066	0.05	0.092	0.048*	0.027*	0.09	0.31
**Persist.**	0.014*	0.028*	0.86	—	0.042*	0.178	0.063	0.049*	0.009*	0.089	0.393
**RandForest**	0.018*	0.073	0.066	0.042*	—	0.886	0.979	0.532	0.487	0.861	0.242
**Calendar**	0.010*	0.046*	0.05	0.178	0.886	—	0.911	0.564	0.618	0.803	0.256
**SARIMAX**	0.052	0.155	0.092	0.063	0.979	0.911	—	0.144	0.4	0.901	0.214
**ARIMAX**	0.056	0.247	0.048*	0.049*	0.532	0.564	0.144	—	0.892	0.676	0.096
**SARIMA**	0.2	0.504	0.027*	0.009*	0.487	0.618	0.4	0.892	—	0.704	0.087
**XGBoost**	0.019*	0.121	0.09	0.089	0.861	0.803	0.901	0.676	0.704	—	0.262
**LSTM**	0.014*	0.025*	0.31	0.393	0.242	0.256	0.214	0.096	0.087	0.262	—

Panel C: 21-day horizon (n = 141 aligned weeks)

Row \ Col	DT-Ad	DT-Base	SeasonalNaive	Persist.	RandForest	Calendar	SARIMAX	ARIMAX	SARIMA	XGBoost	LSTM
**DT-Ad**	—	0.701	0.045*	0.038*	0.127	0.603	0.074	0.177	0.932	0.167	0.368
**DT-Base**	0.701	—	0.030*	0.059	0.158	0.665	0.106	0.236	0.928	0.171	0.321
**SeasonalNaive**	0.045*	0.030*	—	0.495	0.348	0.062	0.471	0.25	0.083	0.352	0.083
**Persist.**	0.038*	0.059	0.495	—	0.15	0.127	0.143	0.101	0.007*	0.146	0.136
**RandForest**	0.127	0.158	0.348	0.15	—	0.402	0.863	0.749	0.272	0.871	0.699
**Calendar**	0.603	0.665	0.062	0.127	0.402	—	0.324	0.56	0.798	0.417	0.596
**SARIMAX**	0.074	0.106	0.471	0.143	0.863	0.324	—	0.047*	0.025*	0.812	0.491
**ARIMAX**	0.177	0.236	0.25	0.101	0.749	0.56	0.047*	—	0.25	0.761	0.881
**SARIMA**	0.932	0.928	0.083	0.007*	0.272	0.798	0.025*	0.25	—	0.27	0.522
**XGBoost**	0.167	0.171	0.352	0.146	0.871	0.417	0.812	0.761	0.27	—	0.711
**LSTM**	0.368	0.321	0.083	0.136	0.699	0.596	0.491	0.881	0.522	0.711	—

Panel D: 28-day horizon (n = 138 aligned weeks)

Row \ Col	DT-Ad	DT-Base	SeasonalNaive	Persist.	RandForest	Calendar	SARIMAX	ARIMAX	SARIMA	XGBoost	LSTM
**DT-Ad**	—	0.951	0.415	0.041*	0.062	0.508	0.732	0.694	0.757	0.187	0.552
**DT-Base**	0.951	—	0.265	0.049*	0.069	0.457	0.623	0.641	0.661	0.131	0.378
**SeasonalNaive**	0.415	0.265	—	0.098	0.601	0.195	0.532	0.25	0.067	0.95	0.041*
**Persist.**	0.041*	0.049*	0.098	—	0.103	0.061	0.074	0.061	0.042*	0.075	0.054
**RandForest**	0.062	0.069	0.601	0.103	—	0.087	0.203	0.107	0.1	0.464	0.099
**Calendar**	0.508	0.457	0.195	0.061	0.087	—	0.355	0.718	0.761	0.087	0.93
**SARIMAX**	0.732	0.623	0.532	0.074	0.203	0.355	—	0.063	0.475	0.416	0.299
**ARIMAX**	0.694	0.641	0.25	0.061	0.107	0.718	0.063	—	0.972	0.152	0.722
**SARIMA**	0.757	0.661	0.067	0.042*	0.1	0.761	0.475	0.972	—	0.173	0.635
**XGBoost**	0.187	0.131	0.95	0.075	0.464	0.087	0.416	0.152	0.173	—	0.12
**LSTM**	0.552	0.378	0.041*	0.054	0.099	0.93	0.299	0.722	0.635	0.12	—

Note: DM test with Newey-West HAC standard errors, loss = MSE. Aligned prediction weeks (n = 147/144/141/138 for 7/14/21/28d) were used for paired comparison. Significance: * $p < 0.05$. DT-Ad (DeformTime-Ad) was never significantly outperformed by any model at any horizon (0 losses across 4 horizons × 10 comparisons).

Supplementary Table S5. Model configuration

Panel A: Parameter and setup configurations of main models

Aspect	DT-Ad	DT-Base	LSTM	XGBoost	Random Forest	SARIMA	ARIMAX	SARIMAX
**Data Granularity**	Weekly	Weekly	Weekly	Weekly	Weekly	Weekly	Weekly	Weekly
**Input Features**	11 CCF-selected + ILI% lag	11 CCF-selected + ILI% lag	11 CCF-selected + ILI% lag	11 CCF-selected + ILI% lag	11 CCF-selected + ILI% lag	ILI% only (no exog)	11 CCF-selected exog	11 CCF-selected exog
**Feature Scaling**	RobustScaler (meteo) + MinMaxScaler (search)	RobustScaler + MinMaxScaler	RobustScaler + MinMaxScaler	RobustScaler + MinMaxScaler	RobustScaler + MinMaxScaler	RobustScaler + MinMaxScaler	RobustScaler + MinMaxScaler	RobustScaler + MinMaxScaler
**Lookback Window**	2 weeks (2 obs)	2 weeks (2 obs)	2 weeks (2 obs)	2 weeks (2 obs)	2 weeks (2 obs)	— (N/A)	— (N/A)	— (N/A)
**Explicit Seasonal Component**	✗	✗	✗	✗	✗	✓ (P,D,Q,52)	✗	✓ (P,D,Q,52)
**Data Split**	3-fold rolling-origin	3-fold rolling-origin	3-fold rolling-origin	3-fold rolling-origin	3-fold rolling-origin	3-fold rolling-origin	3-fold rolling-origin	3-fold rolling-origin
**Hyperparameter Tuning**	Optuna (30 trials, TPE)	— (fixed defaults)	Optuna (30 trials, TPE)	Optuna (30 trials, TPE)	Optuna (30 trials, TPE)	Grid search	Grid search	Grid search
**Sample Weighting***	peak=3.0, active=2.0, non=1.0	— (N/A)	peak=3.0, active=2.0, non=1.0	peak=3.0, active=2.0, non=1.0	peak=3.0, active=2.0, non=1.0	— (N/A)	— (N/A)	— (N/A)

**Weight Thresholds**	mean_train + 1SD / 2SD	mean_train + 1SD / 2SD	mean_train + 1SD / 2SD	mean_train + 1SD / 2SD	mean_train + 1SD / 2SD	mean_train + 1SD / 2SD	mean_train + 1SD / 2SD	mean_train + 1SD / 2SD
**Loss Function**	HuberLoss (δ=1.0)	MSELoss	MSELoss	reg:squarederror	squared_error	MLE (Gaussian)	MLE (Gaussian)	MLE (Gaussian)
**Weight Decay**	1.00E-05	1.00E-05	— (N/A)	— (N/A)	— (N/A)	— (N/A)	— (N/A)	— (N/A)
**Exog Forecast Strategy**	—	—	—	—	—	—	Last-known-value	Last-known-value
**Evaluation Framework**	3-fold rolling-origin × 4 horizons	3-fold rolling-origin × 4 horizons	3-fold rolling-origin × 4 horizons	3-fold rolling-origin × 4 horizons	3-fold rolling-origin × 4 horizons	3-fold rolling-origin × 4 horizons	3-fold rolling-origin × 4 horizons	3-fold rolling-origin × 4 horizons

Panel B: Configurations of Naïve benchmark models

**Aspect**	**Persistence**	**Seasonal-naïve**	**Calendar-based**
**Data Granularity**	Weekly	Weekly	Weekly
**Input Features**	Y(t-1) only	Y(t−52) only	Fourier harmonics (order=3)
**Feature Scaling**	— (N/A)	— (N/A)	— (N/A)
**Lookback Window**	1 week	52 weeks	— (N/A)
**Explicit Seasonal Component**	✗	✓ (lag-52)	✓ (Fourier)
**Data Split**	3-fold rolling-origin	3-fold rolling-origin	3-fold rolling-origin
**Hyperparameter Tuning**	— (N/A)	— (N/A)	— (N/A)
**Sample Weighting**	— (N/A)	— (N/A)	— (N/A)
**Loss Function**	— (N/A)	— (N/A)	MSE (OLS)
**Evaluation Framework**	3-fold rolling-origin × 4 horizons	3-fold rolling-origin × 4 horizons	3-fold rolling-origin × 4 horizons
**Evaluation Metrics**	MAE, sMAPE, MSE	MAE, sMAPE, MSE	MAE, sMAPE, MSE

Note: Sample weighting is applied to DT-Ad (DeformTime-Ad), XGBoost, Random Forest, and LSTM; DeformTime-Base, SARIMA/ARIMAX/SARIMAX, and naïve benchmarks do not support instance-level weighting. Weight thresholds (mean_train + 1SD / 2SD) are computed from each fold's training data and used for stratified evaluation across all models. Optimizer is determined by each model's framework (neural networks: Adam; tree-based: built-in; statistical: MLE) and is not listed as it is an inherent component of each model class rather than a tunable experimental condition. All models are evaluated on the same unweighted metrics to ensure fair comparison. Input format: Supervised models (DT-Ad, DT-Base, LSTM, XGBoost, Random Forest) all use 11 CCF-selected exogenous features plus ILI% lagged values (lookback = 2 weeks). Sequence models (DT-Ad, DT-Base, LSTM) receive input as a [lookback × (11+1)] tensor (11 exogenous features + 1 ILI% per time step); tree-based models (XGBoost, Random Forest) receive a flattened [11 + lookback] vector (11 current exogenous features + 2 lagged ILI% values). SARIMA uses ILI% only; ARIMAX/SARIMAX use 11 exogenous features as exogenous regressors with last-known-value persistence for multi-step forecasting.

Supplementary Table S6. Tuning Budgets

**Model**	**Method**	**Budget**	**Notes**
**DT-Ad**	Optuna TPE	30 trials	Per-fold; early stopping pat.=tuned
**DT-Base**	Fixed default	1	hidden_dim=32, num_heads=8, num_layers=1, lr=1e-4, batch_size=32
**LSTM**	Optuna TPE	30 trials	Per-fold; early stopping pat.=20
**XGBoost**	Optuna TPE	30 trials	Per-fold
**Random Forest**	Optuna TPE	30 trials	Per-fold
**SARIMA**	Grid search	20 combos	5 (p,d,q) × 4 (P,D,Q,s)
**ARIMAX**	Grid search	5 combos	5 (p,d,q); no seasonal component
**SARIMAX**	Grid search	20 combos	5 (p,d,q) × 4 (P,D,Q,s)

Supplementary Table S7. Optuna Search Spaces

**Model**	**Parameter**	**Type**	**Range / Values**
**DT-Ad**	num_heads	categorical	{4, 8}
	hidden_dim	categorical	{32, 64, 128}
	num_layers	int	[1, 2]
	lr	float (log)	[1e-5, 1e-3]
	batch_size	categorical	{16, 32}
	patience	int	[10, 25]
**DT-Base**	hidden_dim	Fixed default	32
	num_heads	Fixed default	8
	num_layers	Fixed default	1
	lr	Fixed default	1.00E-04
	batch_size	Fixed default	32
**LSTM**	hidden_dim	categorical	{32, 64, 96}
	num_layers	int	[1, 3]
	lr	float (log)	[1e-5, 1e-2]
	batch_size	categorical	{16, 32, 64}
	dropout	float	[0.1, 0.5]
**XGBoost**	max_depth	int	[3, 10]
	learning_rate	float (log)	[0.01, 0.3]
	subsample	float	[0.6, 1.0]
	colsample_bytree	float	[0.6, 1.0]
	num_boost_round	fixed	500 (early stopping at 30 rounds)
**Random Forest**	n_estimators	int (step=50)	[100, 500]
	max_depth	int	[5, 20]
	min_samples_split	int	[2, 10]
	min_samples_leaf	int	[1, 5]

Constraint: hidden_dim must be divisible by num_heads and num_groups (num_groups=2).

Supplementary Table S8. SARIMA-family Grid Search Space

Model	Parameter	Range / Values
**SARIMA / SARIMAX**	order (p,d,q)	{(1,0,0), (0,0,1), (1,0,1), (2,0,0), (2,0,1)}
**SARIMA / SARIMAX**	seasonal_order (P,D,Q,s)	{(1,0,1,52), (0,0,1,52), (1,0,0,52), (2,0,1,52)}
**ARIMAX**	order (p,d,q)	{(1,0,0), (0,0,1), (1,0,1), (2,0,0), (2,0,1)}
**ARIMAX**	seasonal_order	None (non-seasonal ARIMA + exogenous)

Supplementary Table S9. Best Hyperparameters per Fold

All hyperparameters were optimized independently within each fold's training set. For each fold, the last year of the training set was held out as a validation set for hyperparameter selection. DeformTime-Base uses fixed defaults (no HPO): hidden_dim=32, num_heads=8, num_layers=1, lr=1e-4, batch_size=32. Naïve benchmarks (Persistence, Seasonal-naïve, Calendar) have no tunable hyperparameters.

Horizon	Fold	Model	num_heads	hidden_dim	num_layers	lr	batch_size	patience	Model	hidden_dim	num_layers	lr	batch_size	dropout
7d	F1	DT-Ad	4	32	1	6.21e-04	16	17	LSTM	32	3	2.50e-03	16	0.185
7d	F2	DT-Ad	8	32	1	6.57e-04	32	24	LSTM	32	1	1.81e-05	64	0.132
7d	F3	DT-Ad	8	64	1	9.25e-04	16	18	LSTM	96	2	2.24e-05	16	0.166
14d	F1	DT-Ad	8	32	1	6.62e-04	16	21	LSTM	96	1	1.06e-04	16	0.253
14d	F2	DT-Ad	4	128	1	8.75e-04	32	25	LSTM	64	1	1.04e-05	64	0.176
14d	F3	DT-Ad	8	32	1	6.86e-04	32	23	LSTM	96	1	9.56e-03	32	0.364
21d	F1	DT-Ad	8	64	2	6.76e-04	32	20	LSTM	64	1	3.63e-05	16	0.36
21d	F2	DT-Ad	4	32	1	6.64e-04	16	23	LSTM	64	2	2.75e-05	64	0.448
21d	F3	DT-Ad	4	32	1	4.94e-04	32	18	LSTM	96	2	9.18e-03	16	0.375
28d	F1	DT-Ad	4	32	1	7.91e-04	32	13	LSTM	96	2	9.17e-03	32	0.152
28d	F2	DT-Ad	4	128	1	4.38e-04	16	15	LSTM	96	1	6.57e-05	64	0.24
28d	F3	DT-Ad	8	128	2	4.19e-04	16	17	LSTM	32	2	4.78e-03	16	0.408

Horizon	Fold	Model	max_depth	learning_rate	subsample	colsample_bytree	Model	max_depth	n_estimators	min_samples_split	min_samples_leaf
7d	F1	XGBoost	10	0.0781	0.974	0.638	Random Forest	20	150	7	4
7d	F2	XGBoost	6	0.0244	0.776	0.659	Random Forest	8	100	7	4
7d	F3	XGBoost	8	0.0147	0.884	1	Random Forest	11	500	10	5
14d	F1	XGBoost	6	0.1778	0.703	0.679	Random Forest	11	100	8	4
14d	F2	XGBoost	10	0.0303	0.811	0.647	Random Forest	11	450	5	1
14d	F3	XGBoost	8	0.0349	0.797	0.838	Random Forest	16	250	2	5
21d	F1	XGBoost	4	0.0475	0.752	0.729	Random Forest	18	450	3	3
21d	F2	XGBoost	9	0.0201	0.607	0.689	Random Forest	5	300	8	2
21d	F3	XGBoost	4	0.1131	0.832	0.723	Random Forest	8	200	7	4
28d	F1	XGBoost	3	0.0178	0.939	0.895	Random Forest	10	150	6	2
28d	F2	XGBoost	6	0.0129	0.81	0.603	Random Forest	12	250	9	1
28d	F3	XGBoost	8	0.2903	0.681	0.753	Random Forest	18	300	6	5

Horizon	Fold	Model	order (p,d,q)	seasonal_order (P,D,Q,s)		order (p,d,q)	Model	order (p,d,q)	seasonal_order (P,D,Q,s)
7d	F1	SARIMA	(0, 0, 1)	(0, 0, 1, 52)	ARIMAX	(0, 0, 1)	SARIMAX	(0, 0, 1)	(2, 0, 1, 52)
7d	F2	SARIMA	(1, 0, 0)	(0, 0, 1, 52)	ARIMAX	(0, 0, 1)	SARIMAX	(0, 0, 1)	(2, 0, 1, 52)
7d	F3	SARIMA	(2, 0, 0)	(2, 0, 1, 52)	ARIMAX	(2, 0, 1)	SARIMAX	(1, 0, 1)	(0, 0, 1, 52)
14d	F1	SARIMA	(0, 0, 1)	(1, 0, 1, 52)	ARIMAX	(0, 0, 1)	SARIMAX	(0, 0, 1)	(2, 0, 1, 52)
14d	F2	SARIMA	(1, 0, 0)	(0, 0, 1, 52)	ARIMAX	(0, 0, 1)	SARIMAX	(0, 0, 1)	(2, 0, 1, 52)
14d	F3	SARIMA	(2, 0, 0)	(2, 0, 1, 52)	ARIMAX	(2, 0, 0)	SARIMAX	(1, 0, 1)	(0, 0, 1, 52)
21d	F1	SARIMA	(0, 0, 1)	(1, 0, 1, 52)	ARIMAX	(0, 0, 1)	SARIMAX	(0, 0, 1)	(2, 0, 1, 52)
21d	F2	SARIMA	(1, 0, 0)	(0, 0, 1, 52)	ARIMAX	(0, 0, 1)	SARIMAX	(0, 0, 1)	(2, 0, 1, 52)
21d	F3	SARIMA	(2, 0, 0)	(2, 0, 1, 52)	ARIMAX	(2, 0, 0)	SARIMAX	(1, 0, 1)	(0, 0, 1, 52)
28d	F1	SARIMA	(0, 0, 1)	(1, 0, 1, 52)	ARIMAX	(0, 0, 1)	SARIMAX	(0, 0, 1)	(2, 0, 1, 52)
28d	F2	SARIMA	(0, 0, 1)	(0, 0, 1, 52)	ARIMAX	(0, 0, 1)	SARIMAX	(0, 0, 1)	(2, 0, 1, 52)
28d	F3	SARIMA	(2, 0, 1)	(2, 0, 1, 52)	ARIMAX	(2, 0, 0)	SARIMAX	(1, 0, 1)	(0, 0, 1, 52)

Note: lr = learning rate; patience = early stopping patience (epochs); num_boost_round fixed at 500 for XGBoost with early stopping at 30 rounds; weight decay = 1e-5 for all DeformTime models; HuberLoss δ = 1.0 for Adapted DeformTime (DT-Ad); gradient clipping max_norm = 1.0 for all neural network models; ReduceLROnPlateau (factor=0.5) for DT-Ad, DeformTime-Base, and LSTM.

Supplementary Table S10. Native-window per-horizon performance (per-fold)

All metrics computed on each model's native evaluation window , with corrections: Persistence uses Y(t−1) to reflect the ~1-week ILI reporting lag; DeformTime predictions are extracted from the horizon-specific output step (no cross-lead-time averaging). Epidemic thresholds are data-driven, computed per fold from the training set (active = mean_train + 1 SD; peak = mean_train + 2 SD), consistent with the main-text Table 8.

Per-Fold Overall Metrics

MAE / sMAPE (%) / MSE / n for each model x horizon x fold. Seasonal-naïve and Calendar-based models are horizon-invariant: their predictions for a given target week depend only on Y(t−52) or Fourier(week number), respectively, and do not change with the forecast horizon; consequently, their metrics are identical across all four horizons. Persistence, by contrast, uses Y(forecast_origin−1), where the forecast origin shifts backward as the horizon lengthens, producing horizon-dependent predictions.

Horizon: 7d (1 week)

Model	Fold 1 MAE	F1 sMAPE	F1 MSE	F1 n	Fold 2 MAE	F2 sMAPE	F2 MSE	F2 n	Fold 3 MAE	F3 sMAPE	F3 MSE	F3 n
Persistence	0.483	12.83	0.491	52	0.581	13.98	0.902	52	0.821	17.64	1.907	52
SeasonalNaive	0.874	25.71	1.361	52	0.999	25.38	2.099	52	0.662	17.46	1.048	52
Calendar	0.575	15.22	0.627	52	0.896	24.16	1.299	52	0.985	23.56	1.782	52
SARIMA	0.560	15.37	0.587	52	0.435	11.68	0.387	52	0.575	12.42	0.999	52
ARIMAX	0.518	14.53	0.508	52	0.729	19.99	0.991	52	0.762	17.28	1.376	52
SARIMAX	0.540	15.27	0.536	52	0.727	19.91	0.999	52	0.724	16.39	1.242	52
XGBoost	0.461	12.31	0.513	49	0.619	19.04	0.494	49	0.632	16.11	0.817	49
Random Forest	0.493	13.40	0.485	49	0.444	12.83	0.376	49	0.566	14.11	0.751	49
LSTM	0.493	13.42	0.537	49	0.828	24.44	1.091	49	0.718	18.29	1.123	49
DT-Base	0.520	14.88	0.489	50	0.656	18.50	0.858	50	0.527	12.21	0.772	50
DT-Ad	0.380	10.69	0.289	50	0.378	10.41	0.331	50	0.501	11.46	0.771	50

Horizon: 14d (2 weeks)

Model	Fold 1 MAE	F1 sMAPE	F1 MSE	F1 n	Fold 2 MAE	F2 sMAPE	F2 MSE	F2 n	Fold 3 MAE	F3 sMAPE	F3 MSE	F3 n
Persistence	0.603	16.41	0.692	52	0.787	18.55	1.452	52	1.059	22.82	2.963	52
SeasonalNaive	0.874	25.71	1.361	52	0.999	25.38	2.099	52	0.662	17.46	1.048	52
Calendar	0.575	15.22	0.627	52	0.896	24.16	1.299	52	0.985	23.56	1.782	52
SARIMA	0.525	14.1	0.596	52	0.568	14.8	0.658	52	0.694	14.95	1.424	52
ARIMAX	0.516	14.3	0.522	52	0.878	23.92	1.503	52	0.827	18.38	1.696	52
SARIMAX	0.546	15.43	0.561	52	0.89	24.26	1.527	52	0.831	18.18	1.674	52
XGBoost	0.477	13.32	0.461	48	0.604	19.1	0.514	48	0.872	22.56	1.23	48
Random Forest	0.566	15.67	0.597	48	0.51	15.66	0.439	48	0.762	18.93	1.208	48
LSTM	0.536	14.65	0.592	48	0.792	24.16	0.889	48	0.868	23.27	1.443	48
DT-Base	0.532	14.8	0.546	49	0.611	18.25	0.572	49	0.612	15.19	0.835	49
DT-Ad	0.444	11.92	0.435	49	0.406	11.47	0.387	49	0.525	13.13	0.597	49

Horizon: 21d (3 weeks)

Model	Fold 1 MAE	F1 sMAPE	F1 MSE	F1 n	Fold 2 MAE	F2 sMAPE	F2 MSE	F2 n	Fold 3 MAE	F3 sMAPE	F3 MSE	F3 n
Persistence	0.563	15.13	0.651	52	0.932	21.64	1.933	52	1.279	27.26	3.934	52
SeasonalNaive	0.874	25.71	1.361	52	0.999	25.38	2.099	52	0.662	17.46	1.048	52
Calendar	0.575	15.22	0.627	52	0.896	24.16	1.299	52	0.985	23.56	1.782	52
SARIMA	0.508	13.74	0.541	52	0.601	15.43	0.812	52	0.85	17.96	1.951	52
ARIMAX	0.519	14.46	0.537	52	1.049	28.09	1.996	52	0.953	21.51	2.245	52
SARIMAX	0.557	15.8	0.618	52	1.079	28.87	2.063	52	0.957	21.52	2.175	52
XGBoost	0.55	14.87	0.708	47	0.608	19.51	0.527	47	0.868	20.94	1.573	47
Random Forest	0.633	17.39	0.86	47	0.478	14.8	0.418	47	0.896	21.85	1.558	47
LSTM	0.541	14.85	0.6	47	0.805	24.87	0.882	47	0.672	17.7	1.079	47
DT-Base	0.591	16.46	0.659	48	0.636	19.69	0.537	48	0.704	18.04	0.999	48
DT-Ad	0.565	15.47	0.64	48	0.445	13.09	0.482	48	0.654	16.46	0.937	48

Horizon: 28d (4 weeks)

Model	Fold 1 MAE	F1 sMAPE	F1 MSE	F1 n	Fold 2 MAE	F2 sMAPE	F2 MSE	F2 n	Fold 3 MAE	F3 sMAPE	F3 MSE	F3 n
Persistence	0.687	18.58	0.844	52	1.08	25.31	2.505	52	1.426	30.36	4.702	52
SeasonalNaive	0.874	25.71	1.361	52	0.999	25.38	2.099	52	0.662	17.46	1.048	52
Calendar	0.575	15.22	0.627	52	0.896	24.16	1.299	52	0.985	23.56	1.782	52
SARIMA	0.521	13.85	0.636	52	1.01	26.52	2.195	52	0.867	19.19	1.648	52
ARIMAX	0.625	17.31	0.73	52	0.885	23.77	1.789	52	0.999	22.87	2.287	52
SARIMAX	0.658	18.66	0.799	52	0.933	25.16	1.886	52	0.987	22.25	2.117	52
XGBoost	0.593	16.59	0.59	46	0.845	25.87	0.913	46	0.821	21.19	1.223	46
Random Forest	0.661	19.06	0.782	46	0.659	20.19	0.656	46	0.916	22.74	1.655	46
LSTM	0.628	17.28	0.651	46	0.719	22.63	0.697	46	0.588	16.73	0.592	46
DT-Base	0.63	17.72	0.771	47	0.688	21.6	0.578	47	0.767	19.85	1.127	47
DT-Ad	0.548	14.9	0.642	47	0.551	16.77	0.537	47	0.814	20.86	1.285	47

Supplementary Table S11. Native-window stratified evaluation by epidemic intensity (per-fold)

MAE / MSE / n for each model × fold × epidemic level. Data-driven thresholds per fold: active = mean_train + 1 SD; peak = mean_train + 2 SD. Fold 1 (2017) had no peak weeks (max ILI% 6.34 < peak threshold 6.385).

Pooled Summary (4-horizon average, mean ± SD across fold × horizon combinations)

Model	PeakMAE	PeakMSE	Peakn	ActiveMAE	ActiveMSE	Activen	Non-epiMAE	Non-epiMSE	Non-epin
RandomForest	2.08±1.01	5.27±4.41	5	1.15±0.73	1.99±1.87	36	0.58±0.15	0.70±0.43	529
DT-Ad	2.12±1.04	5.68±4.39	10	1.01±0.67	1.57±1.42	38	0.45±0.11	0.45±0.19	534
XGBoost	2.31±0.86	6.30±3.56	5	1.13±0.67	1.81±1.58	36	0.62±0.17	0.68±0.37	529
SARIMA	2.42±0.94	7.72±4.97	36	1.42±0.55	2.46±1.62	40	0.47±0.09	0.51±0.29	548
Seasonal-naïve	2.53±1.47	9.61±6.29	36	1.98±0.61	4.90±1.79	40	0.66±0.09	0.77±0.23	548
Persistence	2.78±0.74	10.29±4.74	36	1.80±0.35	3.85±1.09	40	0.67±0.21	1.24±0.92	548
DT-Base	3.13±0.52	10.29±3.14	10	1.45±0.51	2.49±1.41	38	0.52±0.08	0.44±0.12	534
SARIMAX	3.17±0.60	11.54±3.50	36	1.26±0.71	2.22±1.92	40	0.60±0.13	0.64±0.28	548
Calendar	3.18±0.17	10.47±1.11	36	1.29±0.17	2.05±0.58	40	0.63±0.08	0.56±0.03	548
ARIMAX	3.31±0.58	12.51±3.51	36	1.26±0.68	2.18±1.81	40	0.57±0.12	0.57±0.24	548
LSTM	4.42±0.91	20.39±7.90	5	2.02±0.25	4.23±0.92	36	0.56±0.13	0.45±0.16	529

Note: DT-Ad native peak MAE = 2.12 (n = 10) vs DT-Base 3.13 (n = 10), 32.3% lower—consistent with the 31.9% reduction on common target weeks (Table 8). The native-window peak sample (n = 10) is larger than the common-week peak sample (n = 5) because it includes all peak weeks covered by each model's native evaluation window.

Peak Period

Model	Fold	h=7d MAE	h=7d MSE	h=7d n	h=14d MAE	h=14d MSE	h=14d n	h=21d MAE	h=21d MSE	h=21d n	h=28d MAE	h=28d MSE	h=28d n
Persistence	F1	—	—	0	—	—	0	—	—	0	—	—	0
Persistence	F2	1.536	3.369	4	2.4	6.29	4	2.725	8.948	4	3.257	11.965	4
Persistence	F3	2.224	7.143	5	2.822	11.809	5	3.478	15.382	5	3.833	17.41	5
SeasonalNaive	F1	—	—	0	—	—	0	—	—	0	—	—	0
SeasonalNaive	F2	3.9	15.494	4	3.9	15.494	4	3.9	15.494	4	3.9	15.494	4
SeasonalNaive	F3	1.152	3.727	5	1.152	3.727	5	1.152	3.727	5	1.152	3.727	5
Calendar	F1	—	—	0	—	—	0	—	—	0	—	—	0
Calendar	F2	3.016	9.425	4	3.016	9.425	4	3.016	9.425	4	3.016	9.425	4
Calendar	F3	3.341	11.51	5	3.341	11.51	5	3.341	11.51	5	3.341	11.51	5
SARIMA	F1	—	—	0	—	—	0	—	—	0	—	—	0
SARIMA	F2	1.241	2.157	4	1.752	4.167	4	2.357	6.901	4	4.225	18.27	4
SARIMA	F3	1.886	5.06	5	2.072	6.093	5	2.663	8.714	5	3.189	10.358	5
ARIMAX	F1	—	—	0	—	—	0	—	—	0	—	—	0
ARIMAX	F2	2.599	7.603	4	3.442	12.451	4	3.557	14.411	4	4.026	16.517	4
ARIMAX	F3	2.554	8.345	5	2.936	10.217	5	3.288	13.81	5	4.048	16.754	5
SARIMAX	F1	—	—	0	—	—	0	—	—	0	—	—	0
SARIMAX	F2	2.628	7.7	4	3.405	12.26	4	3.565	14.571	4	4.032	16.642	4
SARIMAX	F3	2.297	6.867	5	2.757	8.819	5	3.004	11.469	5	3.699	13.961	5
XGBoost	F1	—	—	0	—	—	0	—	—	0	—	—	0
XGBoost	F2	1.586	2.514	1	—	—	0	—	—	0	—	—	0
XGBoost	F3	1.544	4.027	2	3.093	9.564	1	3.018	9.111	1	—	—	0
Random Forest	F1	—	—	0	—	—	0	—	—	0	—	—	0
Random Forest	F2	1.199	1.438	1	—	—	0	—	—	0	—	—	0
Random Forest	F3	1.332	2.452	2	3.348	11.209	1	2.448	5.993	1	—	—	0
LSTM	F1	—	—	0	—	—	0	—	—	0	—	—	0
LSTM	F2	3.503	12.268	1	—	—	0	—	—	0	—	—	0
LSTM	F3	3.821	15.395	2	5.406	29.227	1	4.966	24.664	1	—	—	0
DT-Base	F1	—	—	0	—	—	0	—	—	0	—	—	0

**DT-Base**	**F2**	3.284	11.291	2	2.611	6.818	1	—	—	0	—	—	0
**DT-Base**	**F3**	2.627	7.496	3	2.839	8.628	2	3.589	12.881	1	3.827	14.648	1
**DT-Ad**	**F1**	—	—	0	—	—	0	—	—	0	—	—	0
**DT-Ad**	**F2**	1.142	1.779	2	0.799	0.639	1	—	—	0	—	—	0
**DT-Ad**	**F3**	2.635	7.526	3	1.871	4.014	2	2.779	7.72	1	3.523	12.409	1

Active Period

Model	Fold	h=7d MAE	h=7d MSE	h=7d n	h=14d MAE	h=14d MSE	h=14d n	h=21d MAE	h=21d MSE	h=21d n	h=28d MAE	h=28d MSE	h=28d n
**Persistence**	**F1**	1.524	2.421	3	1.872	3.554	3	1.949	3.815	3	2.168	4.737	3
**Persistence**	**F2**	1.078	2.463	3	1.429	2.844	3	2.35	5.744	3	2.058	5.47	3
**Persistence**	**F3**	1.533	3.16	4	1.823	4.085	4	1.991	4.412	4	1.764	3.555	4
**SeasonalNaive**	**F1**	2.43	5.981	3	2.43	5.981	3	2.43	5.981	3	2.43	5.981	3
**SeasonalNaive**	**F2**	2.352	6.241	3	2.352	6.241	3	2.352	6.241	3	2.352	6.241	3
**SeasonalNaive**	**F3**	1.155	2.487	4	1.155	2.487	4	1.155	2.487	4	1.155	2.487	4
**Calendar**	**F1**	1.5	2.409	3	1.5	2.409	3	1.5	2.409	3	1.5	2.409	3
**Calendar**	**F2**	1.109	1.259	3	1.109	1.259	3	1.109	1.259	3	1.109	1.259	3
**Calendar**	**F3**	1.272	2.473	4	1.272	2.473	4	1.272	2.473	4	1.272	2.473	4
**SARIMA**	**F1**	1.698	2.924	3	2.008	4.311	3	2	4.026	3	2.25	5.209	3
**SARIMA**	**F2**	0.665	0.637	3	0.985	1.013	3	1.1	1.215	3	1.988	4.298	3
**SARIMA**	**F3**	0.85	0.946	4	1.045	1.379	4	1.004	1.389	4	1.455	2.126	4
**ARIMAX**	**F1**	1.955	4.055	3	1.927	3.901	3	2.14	4.642	3	2.261	5.189	3
**ARIMAX**	**F2**	0.502	0.365	3	0.492	0.362	3	0.848	0.976	3	0.328	0.133	3
**ARIMAX**	**F3**	1.038	1.222	4	1.162	1.557	4	1.002	1.204	4	1.5	2.503	4
**SARIMAX**	**F1**	2.025	4.337	3	1.981	4.118	3	2.195	4.887	3	2.291	5.346	3
**SARIMAX**	**F2**	0.438	0.418	3	0.476	0.435	3	0.807	0.792	3	0.366	0.216	3
**SARIMAX**	**F3**	0.944	1.069	4	1.149	1.473	4	0.973	1.137	4	1.464	2.363	4
**XGBoost**	**F1**	1.814	3.467	3	1.734	3.069	3	2.26	5.167	3	1.718	2.97	3
**XGBoost**	**F2**	0.542	0.317	3	0.438	0.376	3	0.054	0.003	2	1.207	1.481	2
**XGBoost**	**F3**	0.647	0.528	4	0.736	0.837	4	1.015	1.441	3	1.42	2.02	3
**Random Forest**	**F1**	1.603	2.723	3	1.876	3.643	3	2.286	5.317	3	2.262	5.194	3
**Random Forest**	**F2**	0.487	0.557	3	0.332	0.166	3	0.194	0.074	2	0.672	0.498	2
**Random Forest**	**F3**	0.589	0.5	4	1.029	1.411	4	1.374	2.141	3	1.095	1.668	3
**LSTM**	**F1**	2.166	4.709	3	2.262	5.231	3	2.279	5.324	3	2.161	4.845	3
**LSTM**	**F2**	2.15	4.659	3	2.157	4.682	3	2.125	4.555	2	1.852	3.454	2
**LSTM**	**F3**	1.413	2.117	4	1.981	4.049	4	1.828	3.432	3	1.891	3.658	3
**DT-Base**	**F1**	1.846	3.641	3	1.688	3.089	3	1.993	4.077	3	2.32	5.412	3
**DT-Base**	**F2**	1.329	1.808	3	1.315	1.744	3	1.485	2.221	3	1.3	1.702	2
**DT-Base**	**F3**	0.472	0.412	4	0.784	0.958	4	1.176	1.772	4	1.724	3.08	3
**DT-Ad**	**F1**	1.369	2.167	3	1.485	2.298	3	1.798	3.283	3	1.933	3.764	3
**DT-Ad**	**F2**	0.409	0.271	3	0.386	0.16	3	0.098	0.013	3	0.15	0.033	2
**DT-Ad**	**F3**	0.565	0.532	4	0.976	1.118	4	1.127	1.705	4	1.786	3.55	3

Non-epidemic Period

Model	Fold	h=7d MAE	h=7d MSE	h=7d n	h=14d MAE	h=14d MSE	h=14d n	h=21d MAE	h=21d MSE	h=21d n	h=28d MAE	h=28d MSE	h=28d n
**Persistence**	**F1**	0.419	0.373	49	0.526	0.517	49	0.478	0.458	49	0.596	0.606	49
**Persistence**	**F2**	0.463	0.578	45	0.601	0.929	45	0.679	1.055	45	0.822	1.467	45
**Persistence**	**F3**	0.591	1.182	43	0.783	1.83	43	0.957	2.558	43	1.115	3.331	43
**SeasonalNaive**	**F1**	0.778	1.078	49	0.778	1.078	49	0.778	1.078	49	0.778	1.078	49
**SeasonalNaive**	**F2**	0.651	0.632	45	0.651	0.632	45	0.651	0.632	45	0.651	0.632	45
**SeasonalNaive**	**F3**	0.559	0.603	43	0.559	0.603	43	0.559	0.603	43	0.559	0.603	43
**Calendar**	**F1**	0.518	0.518	49	0.518	0.518	49	0.518	0.518	49	0.518	0.518	49
**Calendar**	**F2**	0.694	0.58	45	0.694	0.58	45	0.694	0.58	45	0.694	0.58	45
**Calendar**	**F3**	0.684	0.587	43	0.684	0.587	43	0.684	0.587	43	0.684	0.587	43
**SARIMA**	**F1**	0.491	0.444	49	0.434	0.369	49	0.417	0.328	49	0.415	0.356	49
**SARIMA**	**F2**	0.348	0.213	45	0.435	0.323	45	0.412	0.244	45	0.66	0.626	45
**SARIMA**	**F3**	0.397	0.532	43	0.501	0.885	43	0.625	1.216	43	0.542	0.59	43
**ARIMAX**	**F1**	0.43	0.291	49	0.43	0.315	49	0.42	0.286	49	0.525	0.457	49
**ARIMAX**	**F2**	0.578	0.445	45	0.675	0.606	45	0.84	0.96	45	0.643	0.59	45
**ARIMAX**	**F3**	0.528	0.58	43	0.551	0.718	43	0.677	0.997	43	0.598	0.585	43
**SARIMAX**	**F1**	0.449	0.303	49	0.458	0.343	49	0.457	0.357	49	0.558	0.52	49
**SARIMAX**	**F2**	0.577	0.442	45	0.694	0.646	45	0.876	1.036	45	0.695	0.685	45
**SARIMAX**	**F3**	0.521	0.604	43	0.578	0.862	43	0.718	1.192	43	0.628	0.716	43
**XGBoost**	**F1**	0.373	0.32	46	0.394	0.287	45	0.433	0.403	44	0.514	0.424	43
**XGBoost**	**F2**	0.603	0.461	45	0.615	0.523	45	0.633	0.55	45	0.828	0.888	44
**XGBoost**	**F3**	0.588	0.694	43	0.833	1.072	43	0.808	1.407	43	0.779	1.168	43
**Random Forest**	**F1**	0.421	0.339	46	0.479	0.394	45	0.52	0.556	44	0.549	0.474	43
**Random Forest**	**F2**	0.424	0.34	45	0.522	0.457	45	0.491	0.433	45	0.659	0.664	44
**Random Forest**	**F3**	0.528	0.695	43	0.677	0.956	43	0.827	1.415	43	0.903	1.655	43
**LSTM**	**F1**	0.384	0.265	46	0.421	0.282	45	0.422	0.278	44	0.522	0.359	43
**LSTM**	**F2**	0.681	0.605	45	0.701	0.636	45	0.746	0.719	45	0.667	0.572	44
**LSTM**	**F3**	0.509	0.367	43	0.66	0.554	43	0.491	0.367	43	0.497	0.378	43
**DT-Base**	**F1**	0.435	0.287	47	0.456	0.381	46	0.498	0.431	45	0.514	0.455	44
**DT-Base**	**F2**	0.495	0.331	45	0.52	0.355	45	0.58	0.425	45	0.661	0.528	45
**DT-Base**	**F3**	0.385	0.336	43	0.493	0.461	43	0.594	0.651	43	0.629	0.677	43
**DT-Ad**	**F1**	0.317	0.169	47	0.376	0.314	46	0.483	0.464	45	0.454	0.429	44
**DT-Ad**	**F2**	0.342	0.271	45	0.398	0.397	45	0.468	0.514	45	0.569	0.559	45
**DT-Ad**	**F3**	0.346	0.322	43	0.421	0.389	43	0.561	0.708	43	0.684	0.869	43

Note: '—' indicates no predictions available for that fold/horizon/level combination. For supervised models (XGBoost, Random Forest, LSTM), the lookback+horizon burn-in excludes early-year peak weeks at longer horizons, resulting in n = 0 for the peak stratum at 28d. Persistence uses Y(t−1); DeformTime uses horizon-specific extraction. Native-window values differ from the common-target-week main-text Tables 6 and 8.

Supplementary Table S12. Three-fold rolling-origin

Analytical Step	Fold 1	Fold 2	Fold 3
**CCF feature selection**	2010–2016(frozen, all folds)	—	—
**PCA fitting**	2010–2016(frozen, all folds)	—	—
**Scaler fit(RobustScaler/MinMaxScaler)**	2010–2016	2010–2017	2010–2018
**Epidemic threshold calc**	2010–2016	2010–2017	2010–2018
**Sample weight calc**	2010–2016	2010–2017	2010–2018
**HPO search**	2010–2015	2010–2016	2010–2017
**HPO validation**	2016	2017	2018
**Model fitting**	2010–2016	2010–2017	2010–2018
**Test (untouched)**	2017	2018	2019

Within each fold, the last year of the training set was held out for hyperparameter validation, and all data-dependent procedures (scaling, threshold estimation, sample weighting, and model training) were performed exclusively on the training portion of that fold. CCF feature selection and PCA fitting were performed once on the Fold 1 training set (2010–2016) and frozen across all folds. CCF uses both the predictor variables X and the target Y (ILI%), but only within the 2010–2016 Fold 1 training period; because the selected features are frozen across all subsequent folds, the test years (2017–2019) are never accessed, precluding test-set leakage. PCA fitting is unsupervised (using only predictor variables X, not the target Y), introducing no target leakage.

Supplementary Table S13. Ablation study per-horizon performance (common target weeks).

Variant	Modification	7dMAE	7dMSE	14dMAE	14dMSE	21dMAE	21dMSE	28dMAE	28dMSE
**Full(DeformTime-Ad)**	—	0.392	0.394	0.451	0.468	0.561	0.699	0.611	0.732
**Abl-1(noConv1d-offset)**	Linear-offsetwithgather	0.434	0.442	0.484	0.548	0.588	0.732	0.63	0.751
**Abl-2(noHPO)**	Fixeddefaulthyperparameters	0.823	1.153	0.575	0.72	0.712	0.883	0.605	0.727
**Abl-3(noweighting)**	Uniformsampleweights	0.411	0.413	0.443	0.443	0.576	0.68	0.598	0.686
**Abl-4(MSEloss)**	MSEreplacesHuberloss	0.417	0.429	0.473	0.528	0.595	0.729	0.577	0.697
**Abl-5(meteo-only)**	6meteorologicalfeaturesonly	0.424	0.461	0.475	0.545	0.57	0.698	0.592	0.685
**Abl-6(search-only)**	5searchfeaturesonly	0.422	0.428	0.598	0.748	0.603	0.759	0.612	0.734

Note: Common target weeks (n = 147/144/141/138 for 7/14/21/28d). DeformTime predictions use horizon-specific extraction (j = H−1). 4-horizon averages (mean ± SD across 3 folds × 4 horizons) are reported in Table 9. Abl-2 (no HPO) shows the most severe degradation at 7d (MSE 1.153 vs. 0.394 for Full). Abl-6 (search-only) degrades sharply at 14–28d, while Abl-5 (meteo-only) remains competitive at longer horizons, reflecting the lead-time advantage of meteorological variables.
